# Musculoskeletal practitioners’ perceptions of contextual factors that may influence chronic low back pain outcomes: a modified Delphi study

**DOI:** 10.1186/s12998-023-00482-4

**Published:** 2023-04-05

**Authors:** Bronwyn Sherriff, Carol Clark, Clare Killingback, Dave Newell

**Affiliations:** 1 Department of Rehabilitation and Sport Sciences, Faculty of Health and Social Sciences, Bournemouth University, 504 Bournemouth Gateway Building, 10 St Paul’s Lane, Bournemouth, Dorset BH8 8AJ England; 2 AECC University College, Bournemouth, England; 3 Department of Sport, Health and Exercise Sciences, Faculty of Health Sciences, University of Hull, Hull, England

**Keywords:** Chronic low back pain, Musculoskeletal pain, Contextual factors, Delphi study, Placebo effect, Physician-patient relations, Health professional-patient relations, Physiotherapy, Chiropractic, Osteopathy

## Abstract

**Background:**

Optimal shaping of contextual factors (CFs) during clinical encounters may be associated with analgesic responses in treatments for musculoskeletal pain. These CFs (i.e., the patient-practitioner relationship, patient’s and practitioner’s beliefs/characteristics, treatment characteristics, and environment) have not been widely evaluated by musculoskeletal practitioners. Understanding their views has the potential to improve treatment quality and effectiveness. Drawing on a panel of United Kingdom practitioners’ expertise, this study aimed to investigate their perceptions of CFs during the management of patients presenting with chronic low back pain (LBP).

**Methods:**

A modified two-round online Delphi-consensus survey was conducted to measure the extent of panel agreement regarding the perceived acceptability and influence of five main types of CFs during clinical management of patients with chronic LBP. Qualified musculoskeletal practitioners in the United Kingdom providing regular treatment for patients with chronic LBP were invited to take part.

**Results:**

The successive Delphi rounds included 39 and 23 panellists with an average of 19.9 and 21.3 years of clinical experience respectively. The panel demonstrated a high degree of consensus regarding approaches to enhance the patient-practitioner relationship (18/19 statements); leverage their own characteristics/beliefs (10/11 statements); modify the patient’s beliefs and consider patient’s characteristics (21/25 statements) to influence patient outcomes during chronic LBP rehabilitation. There was a lower degree of consensus regarding the influence and use of approaches related to the treatment characteristics (6/12 statements) and treatment environment (3/7 statements), and these CFs were viewed as the least important. The patient-practitioner relationship was rated as the most important CF, although the panel were not entirely confident in managing a range of patients’ cognitive and emotional needs.

**Conclusion:**

This Delphi study provides initial insights regarding a panel of musculoskeletal practitioners’ attitudes towards CFs during chronic LBP rehabilitation in the United Kingdom. All five CF domains were perceived as capable of influencing patient outcomes, with the patient-practitioner relationship being perceived as the most important CF during routine clinical practice. Musculoskeletal practitioners may require further training to enhance their proficiency and confidence in applying essential psychosocial skills to address the complex needs of patients with chronic LBP.

**Supplementary Information:**

The online version contains supplementary material available at 10.1186/s12998-023-00482-4.

## Background

Healthcare practitioners’ views regarding the recognition and modulation of contextual factors (CFs) during routine clinical practice is important and has the potential to improve the quality and effectiveness of patient care [1, 2]. CFs are integral to both placebo and nocebo effects, capable of triggering positive or negative clinical outcomes, particularly in their capacity to modulate patients’ pain [1, 3]. One categorisation of CFs encompasses five broad domains: (i) the patient-practitioner relationship; (ii) patient’s characteristics/beliefs; (iii) practitioner’s characteristics/beliefs; (iv) the treatment characteristics; and (v) the treatment environment/setting [4]. These CFs are conceptualised to include the patient’s perception of both the external context such as the healthcare environment, treatment, and associated social cues, (e.g., verbal suggestions, practitioner features) together with their internal context such as their prior experiences, emotional states, and expectations which then mutually informs their appraisal of future health and wellbeing [1, 3].

CF mediated pain modulation involves defined endogenous neural pathways evoked by psychological processes such as a patient’s mindset, expectations, or social and observational learning [5–7]. Both the social and environmental features of the treatment context inform these psychological processes, which are conscious and non-conscious. The mindset of a patient regarding their health, specific illness, and treatment is also influenced by the patient-practitioner relationship which affects both the quality and effectiveness of care received [5, 7, 8]. Accordingly, healthcare practitioners are capable of shaping patients’ thoughts and feelings during therapeutic encounters via (a) cognitive care—influencing patients’ expectations regarding their treatment or illness beliefs; and (b) emotional care—influencing unhelpful emotional states (e.g., fear, anxiety) through empathy, warmth, and reassurance [4]. In the context of health and illness, dyadic interactions between patients and practitioners serve as a conduit for exchanging sociobiological information [5]. Developing a positive therapeutic alliance or a person-centred approach creates a foundation for interpersonal healing which can either catalyse or inhibit placebo and nocebo effects respectively. How practitioners establish the recovery context can positively shape patients’ expectations and influence their clinical outcomes [5, 6]. Optimal shaping of CFs during clinical encounters may be associated with substantive placebo effects such as pain reduction; conversely, a negative treatment environment may be associated with nocebo effects, potentially increasing pain [9]. The patient-practitioner relationship, environmental and social cues, and even the observation of others can add to or stimulate placebo/nocebo effects [3, 5, 6]. The experience and magnitude of such effects is modulated by an individual’s psychosocial perceptions, whether positive or negative, which arises from the context in which they occur [3, 5, 10, 11].

A proposed range of clinical applications to potentially harness placebo effects for non-malignant pain was categorised using the five main CF domains [12]. The authors examined 169 studies derived from seven systematic reviews relating to placebo literature across a range of settings. The initial list was evaluated and validated by leading placebo researchers using a survey, resulting in a taxonomy of possible clinical applications to deliberately harness placebo effects during routine practice [12]. Similarly, other clinicians and researchers have also recommended approaches to avoid nocebo effects [13] and enhance placebo effects for pain and musculoskeletal (MSK) disorders [14, 15]. This raises the possibility of ethically harnessing placebo analgesia and integrating such effects into clinical rehabilitation, particularly for MSK pain.

It is important to note that the aforementioned applications originate from a range of studies that may include healthy controls, experimental designs, or have been extrapolated from qualitative research [1, 14, 15]. Accordingly, it is yet to be explicitly uncovered how CFs may be optimally or consistently harnessed to induce placebo analgesia during clinical practice for specific MSK conditions. Moreover, during MSK rehabilitation, predictions in clinical practice may be challenging since disentangling effects underpinned by CFs, effects of complex interventions with interacting components, and confounding factors (e.g., natural history, symptom regression to the mean) is complicated [15, 16]. There is growing recognition that translational placebo research is required [1, 17] to explore and understand patients’, practitioners’, and other stakeholders’ views regarding the ethical and appropriate use of CFs for different MSK disorders, as well as for acute and chronic conditions [1, 12, 15, 17].

Recently, a national Italian survey examining manual therapists’ (MTs) perspectives regarding the use of CFs during clinical practice [2] and a subsequent investigation of Italian physiotherapists’ views [18] suggest these practitioners believe CFs contribute to therapeutic effects. However, neither focused on the relevance of CFs in relation to a specific MSK condition. Since there are numerous placebo/nocebo effects with distinctive mechanisms across a range of illnesses and interventions [19, 20], it is important to investigate practitioners’ attitudes towards the use of CFs for particular health complaints.

MSK conditions account for a considerable proportion of persistent pain globally [21, 22] with low back pain (LBP) being a leading cause of disability [23–26] particularly in regions with higher life expectancies [27]. The prevalence of chronic LBP (i.e., persistent symptoms for 12 or more weeks) is approximately 19.6% between the economically active ages of 20 and 59 years [28]. Persistent LBP negatively impacts patients’ quality of life, activity levels, ability to work, and earning potential [27] creating deleterious personal, social, and economic consequences [29–31]. Existing chronic LBP (cLBP) treatments are inadequate [32], and those focusing on symptom management typically provide modest relief [31, 33, 34]. Consequently, multimodal cLBP management strategies incorporating the biopsychosocial perspective are required [32].

There is an opportunity to harness placebo effects and clinical practices which involve social and cognitive pain modulation [35] to improve treatment effectiveness for patients with cLBP [32]. Understanding MSK practitioners’ beliefs regarding the deliberate use of CFs during cLBP management may identify areas for further training and skills development. Consequently, there is a need for studies on CFs to support clinicians in implementing contemporary research knowledge in everyday practice [1, 17, 36, 37]. It is unclear whether MSK practitioners believe they have sufficient skills or knowledge to incorporate them into clinical practice which may present a barrier for implementation. Accordingly, it is important to understand practitioners’ views to determine whether there is collective agreement on which of these CF care approaches are perceived as clinically valid or appropriate for the management of cLBP. Drawing on United Kingdom (UK) MSK practitioners’ collective opinions and knowledge, may help understand the present appetite for the modulation of CFs which are perceived to augment usual care for patients with cLBP and the identification of further potentially effective CFs for further study.

## Materials and methods

### Aims

The primary aim of this study was to explore a panel of UK MSK practitioners’ perceptions regarding the acceptability and influence of five main types of CFs during clinical management of patients with cLBP using an iterative process to determine whether group-level consensus was reached. Accordingly, the primary research questions are: (a) To what extent do a panel of UK MSK practitioners perceive CFs as clinically acceptable care approaches capable of influencing patients cLBP outcomes? And (b) To what extent do the panellists agree with each other regarding the use of CF care approaches to influence clinical outcomes for patients with cLBP? Secondary research questions explore the extent to which the UK panel use and regard CFs as clinically valid and important, and how confident they are in applying CFs during the routine care of patients with cLBP. To clarify, the objective of this Delphi study is not to provide recommendations regarding which CFs are important, nor to prescribe their use by other healthcare practitioners.

### Research design

This study involved a modified two-round online Delphi-consensus survey to achieve panel consensus following recommendations for conducting and reporting Delphi studies (CREDES) in palliative medicine where appropriate [38]. Similar methods were used to achieve consensus amongst prominent interdisciplinary placebo researchers regarding the ethical use of placebo/nocebo effects during clinical practice [36], to ascertain what should be disclosed to patients, and how practitioners should be trained [37].

**Fig. 1:**
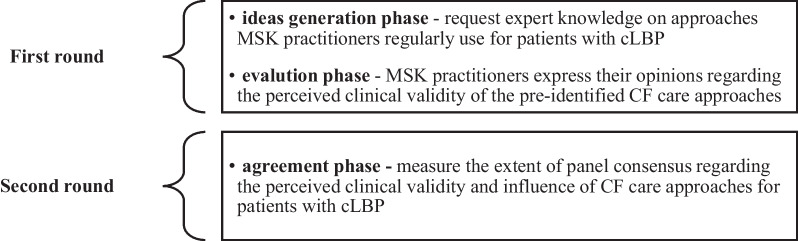
Purpose of each Delphi round

The Delphi-method is a structured group-approach, involving anonymous experts, with the objective of iteratively reducing the range of responses to measure consensus [39]. Compared to the nominal group technique, structured group meetings using an experienced moderator are not necessary enabling broader geographical inclusion [40], encouraging honest and open expression of opinions, and reducing the likelihood of dominant ideas, group pressure or social conformity which can potentially confound the results [41, 42]. The number of rounds was decided a priori since attrition may increase following successive iterations [40, 43]. Consequently, the ideas generation and evaluation phases [39] were combined rather than conducting three rounds. The between-round aims were to refine, clarify and reduce redundant statements whilst including panel suggestions [44]. Incorporating pre-determined content derived from literature reviews, guidelines or preparatory work is another accepted Delphi study modification [44]. The purpose of each iteration is presented in Fig. 1 below.

### Participants

This study aimed to recruit between 20 and 40 qualified UK MSK practitioners assuming a 25% drop-out rate between rounds (i.e., 15–30 panellists in the last round). This is consistent with a systematic review indicating 64% of Delphi studies had between 11 and 50 participants in the final round [45]. The aim was to recruit a heterogenous group of MSK practitioners with an interest in the study as the purpose, resources, and complexity determine an appropriate panel size [46, 47]. Although there are no clear rules regarding panel selection and size [42], convenience or purposive samples are frequently used [44].

This Delphi study used convenience sampling as potential participants were identified and recruited using direct emails through publicly listed websites (e.g., Chartered Society of Physiotherapy, British Chiropractic Association, General Chiropractic Council, BackCare charity) and social media advertisements (e.g., Musculoskeletal Association of Chartered Physiotherapists Twitter page; Understanding Placebo Effects in Manual Therapy Facebook group). Email invitations were also sent via professional networks and word-of-mouth recommendations (i.e., snowballing). Although National Health Service (NHS) practitioners were not directly targeted, five panellists provided personal email addresses during the first-round.

Participants required at least three years of clinical experience which appears to be a common admission requirement for UK master’s training. Since CFs represent psychosocial aspects of care, it was important to include recently qualified MSK practitioners who may have exposure to biopsychosocial training. Panellists therefore self-identified as MSK ‘experts’, proficient in the rehabilitation of patients with cLBP, based on inclusion–exclusion criteria presented in Table 1 below.

**Table 1:** Eligibility criteria

Inclusion criteria	Exclusion criteria
Qualified Physiotherapists, Chiropractors, Osteopaths, or Sports Therapists	Non-qualified/student manual and physical therapists
Three or more years’ clinical experience in providing regular care for patients with cLBP	Fewer than three years’ clinical experience in providing regular care for patients with cLBP
Currently practising in the United Kingdom	Practising outside the United Kingdom or healthcare practitioners who do not primarily provide manual and physical therapy (e.g., General Practitioners, Psychologists, Orthopaedic surgeons)
Able and willing to respond to an online survey in English	

### Materials: survey development and piloting

Preliminary Delphi statements were extracted from various researchers’ recommendations for potentially harnessing placebo effects during clinical practice and relevant reviews [1, 12–15, 48–54]. The first-round survey was initially developed and piloted with two independent/non-participating Physiotherapists and a Chiropractor providing input concerning: time taken to complete; overall clarity, language, terminology/phrasing; ease of completion (e.g., layout, instructions); general comments and functionality. Following ethics approval, participants were invited to complete the first-round survey. Thereafter, the second-round Delphi survey was modified and piloted (*n* = 5). Two non-participating Physiotherapists, a Chiropractor, a professor familiar with Delphi studies and survey design, along with an academic who has previously published research relating to CFs critically evaluated the survey to ensure face and content validity. To review the modifications to the survey between rounds, please refer to Additional file 1: Tables S1 and S2 respectively.

### Data collection procedure

Bournemouth University’s (England) Research Ethics Panel provided ethics approval prior to data collection (**IDs**: **28052** and **32406**, approved on 30/10/2019 and 18/06/2020 for each version of the questionnaire respectively). Data were collected over encrypted SSL (TLS) connections via the JISC online survey platform (https://www.jisc.ac.uk/online-surveys) following informed consent, from 13 January until 11 March 2020 and from 23 June until 23 July 2020 for each round respectively.

**Fig. 2:**
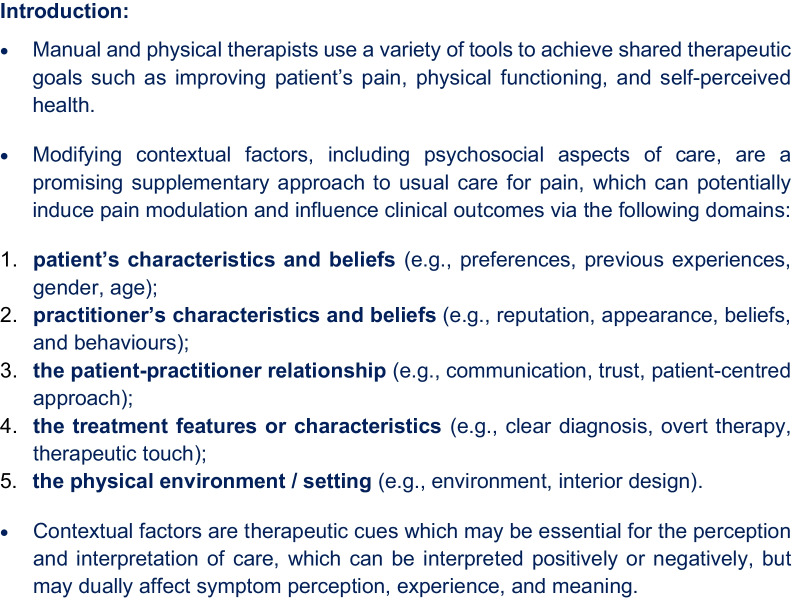
Copy of the introductory text preceding the first-round survey questions

In total, 64 statements were included in the first round, accompanied by open-ended questions so panellists could provide ideas for each of the five main CF domains. A brief introduction was included, to ensure there was a general understanding of the topic, with verbatim text presented in Fig. 2 below.

**Fig. 3:**
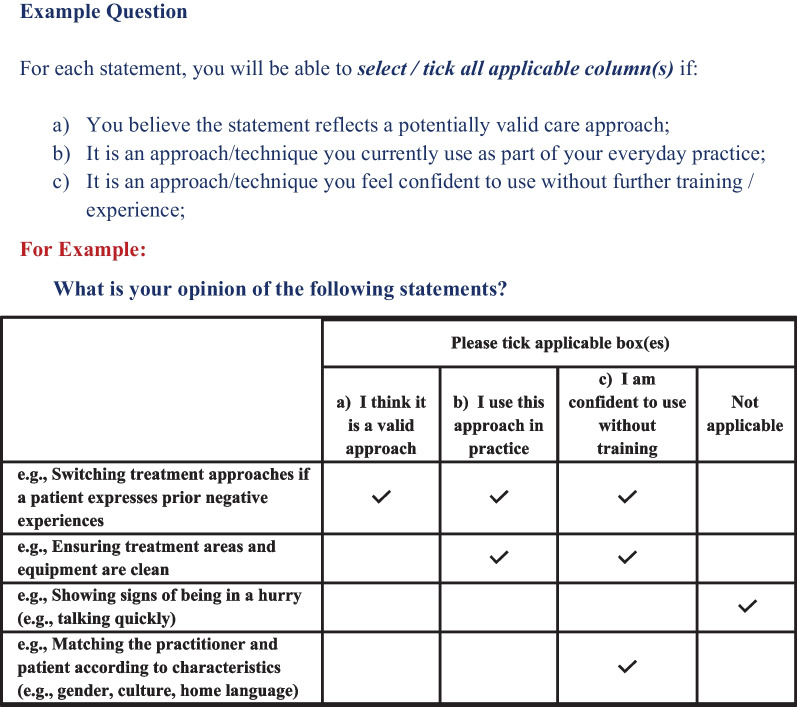
Example question and response options included in the first-round survey

Panellists were asked to “*select/tick all applicable column(s)*” if they believed the corresponding statement: (a) reflected a potentially valid care approach; (b) is an approach they currently use as part of their everyday practice; and (c) is an approach they feel confident to use without further training/experience; or alternatively, they believed the corresponding care approach might contribute to or enhance overall treatment effects. An example of the question format was included to ensure the instructions were clear and easy to follow, as depicted in Fig. 3 below.

During the first round, panellists did not rate their agreement levels nor indicate the strength of their preference, they simply selected the applicable response option(s) as depicted in Fig. 3 above. The final section of the survey included basic demographic information (i.e., age, gender, practitioner type, practice setting, and region), and an option to provide their email address for second-round participation. Panellists expressing interest during the first round were subsequently invited to participate in the second-round (*n* = 31).

During the second round, demographic data were collected first. Thereafter, panellists rated 74 statements using a five-point Likert scale ranging from strongly disagree (1) to strongly agree (5) to indicate whether they had intentionally used each CF approach believing it could influence cLBP outcomes. Two additional response options (i.e., *Not Valid*, and *Do Not Recall/Use—*coded as 0 and missing respectively) were provided which is appropriate where participants have varied knowledge or qualifications [43]. The following instructions preceded each set of statements:

- Below is a list of care approaches for patients with chronic or persistent low back pain (LBP).
- Please indicate whether you **have intentionally used **each approach **believing it could influence patient’s LBP outcome(s)**.

**Fig. 4:**
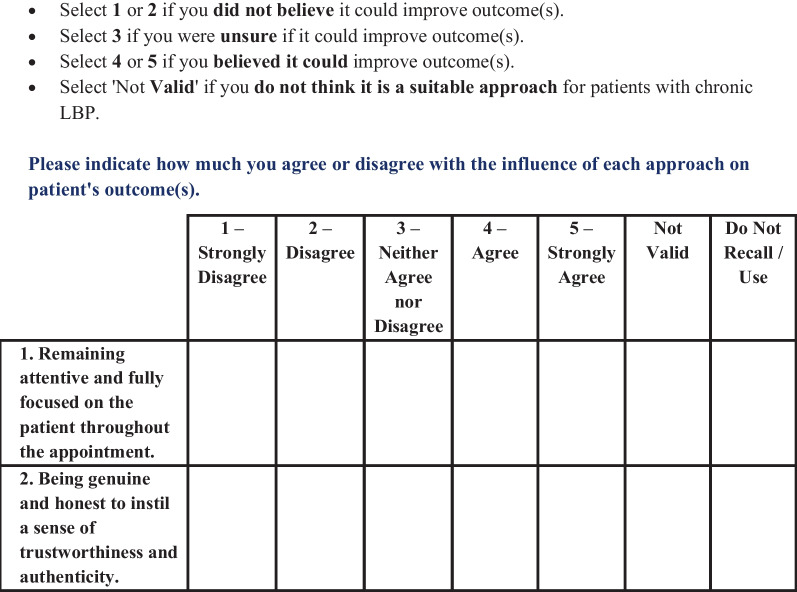
Example of the question format and response options during the second-round survey

Panellists were then asked to indicate the extent to which they agreed or disagreed with the influence of each CF approach on patients’ cLBP outcome(s) as depicted in Fig. 4 below.

To review original copies of each round of the Delphi survey, refer to Additional file 1: DS-R1 and DS-R2 respectively.

### Data analysis

The main analysis involved generating descriptive statistics and frequency tables using SPSS version 28.0. Mean scores were used to rank statements for each of the five main CF domains. Consensus was defined a priori as percentage agreement ≥ 75% (i.e., panellists rating 4 or 5) except if a panellist disagreed (i.e., ratings of 1 or 2) or rated the statement as ‘*Not Valid*’ (0) during the second round. Cumulative percentages were calculated to measure overall panel agreement (i.e., ratings ≥ 4) for each statement.

## Results

### Response rates

**Fig. 5:**
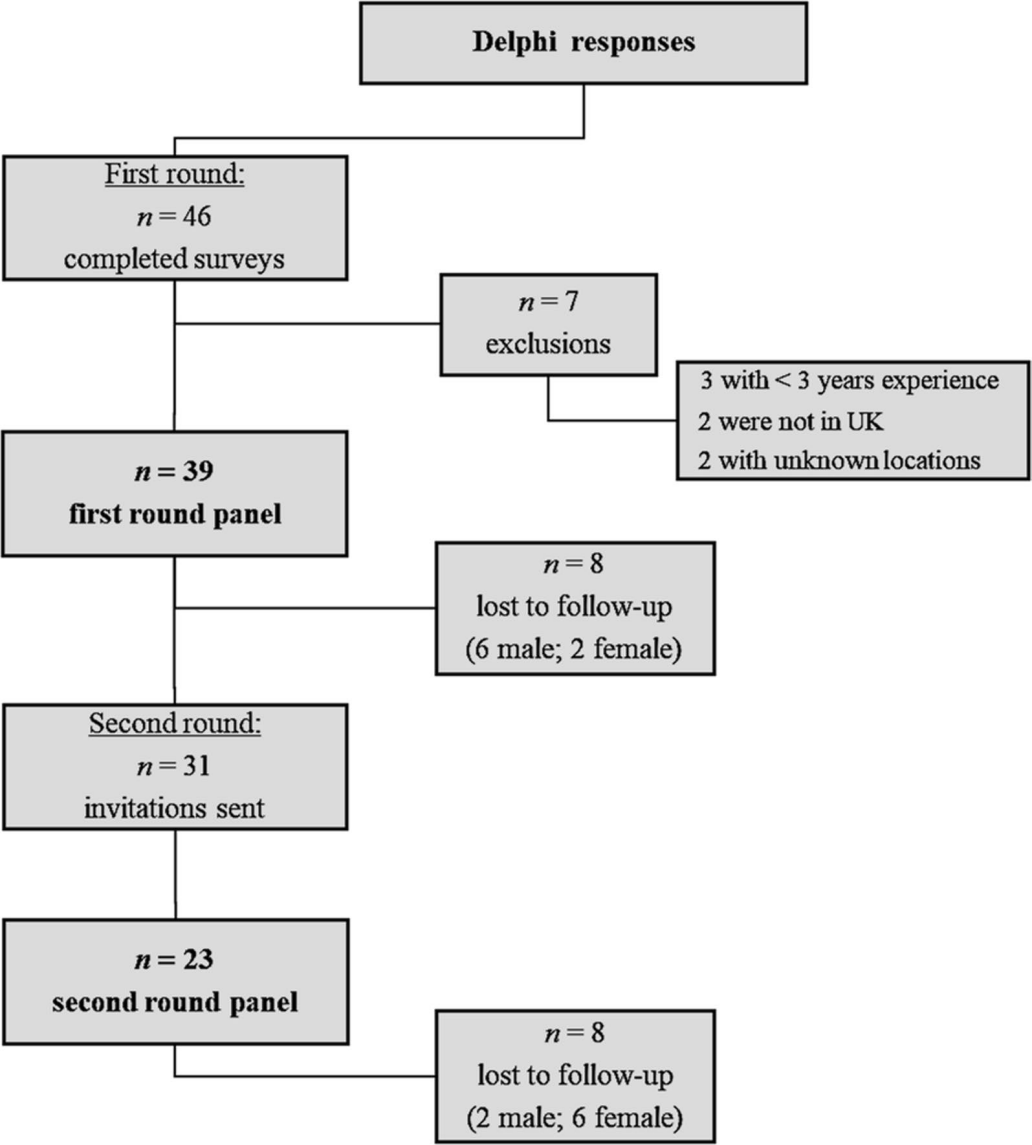
Flowchart of Delphi responses

The first-round panel consisted of 39 qualified MSK practitioners in the UK. Thirty-one practitioners expressed interest in the second round, whilst eight did not. Thus, the attrition rate was 25.8% (i.e., 8/31) between the two iterations. Of the 31 invitations sent, another eight were lost to follow-up as depicted in Fig. 5 below. The second-round response rate was 74.2% (i.e., 23/31) with an overall attrition rate from the original sample of 41.0% (i.e., 16/39).

### Panel characteristics

During the first round (*n* = 39), more than half the panel were male MSK practitioners (56.4%; *n* = 22). Overall, the average age was 46.5 years (*S.D.* ± 11.7; range: 28–75 years), with an average of 19.9 years of clinical experience (*S.D*. ± 10.3; range: 3–40 years). During the second round (*n* = 23), the majority of the panel were also male MSK practitioners (60.9%; *n* = 14). The average age was 47.9 years (*S.D.* ± 11.9; range: 32–75 years) with an average of 21.3 years of clinical experience (*S.D*. ± 11.5; range: 3–41 years). Table 2 below presents a summary of the panel’s characteristics for each round.

**Table 2:** Summary of panel’s characteristics

Demographic information	Round 1 (*n* = 39)		Round 2 (*n* = 23)		Total dropouts (%)
	Frequency	%	Frequency	%	
*Gender*					
Male	22	56.4	14	60.9	8 (20.5)
Female	17	43.6	9	39.1	8 (20.5)
*Practitioner type*					
Chiropractor	23	59.0	16	69.6	7 (17.9)
Physiotherapist	10	25.6	4	17.4	6 (15.4)
Osteopath	4	10.3	3	13.0	1 (2.6)
Other^a^	2	5.1	0	0	2 (5.1)
*Practice setting*					
Private practice	28	71.8	18	78.3	10 (25.6)
Public (NHS)	5	12.8	4	17.4	1 (2.6)
Combination	3	7.7	0	0	3 (7.7)
Other^b^	3	7.7	1	4.3	2 (5.1)
*Practice region*					
South West	10	25.6	7	30.4	3 (7.7)
London	6	15.4	2	8.7	4 (10.3)
South East	6	15.4	4	17.4	2 (5.1)
Wales	5	12.8	4	17.4	1 (2.6)
Scotland	3	7.7	1	4.3	2 (5.1)
East Midlands	3	7.7	1	4.3	2 (5.1)
Yorkshire and the Humber	2	5.1	2	8.7	0 (0)
Northern Ireland	1	2.6	0	0	1 (2.6)
North East and Cumbria	1	2.6	0	0	1 (2.6)
North West	1	2.6	1	4.3	0 (0)
West Midlands	1	2.6	1	4.3	0 (0)

^a^Other practitioners: Chiropractor and Physiotherapist; Clinical Functional Neurologist registered as a Chiropractor

^b^Other settings: Round 1: Private practice and education; education and charity sector; combination of private practice and corporate/manufacturing sectors; Round 2: Educational organisation

### Self-reported use and perceived influence of CFs

Tables 3, 5, 7, 9 and 11 below describe the panel’s (*n* = 39) self-reported use of the 64 statements under consideration during the first round, and their perceptions regarding the clinical validity or appropriateness of these CF care approaches for patients presenting with cLBP. Furthermore, Tables 4, 6, 8, 10 and 12 present the panel’s (*n* = 23) agreement levels with each of the 74 statements under consideration during the second round along with indicating the panel’s consensus (i.e., their agreement with each other) regarding the perceived influence of each statement during the treatment of patients with cLBP. Across each of the five main CF domains, statements have been ranked using the Likert-score mean. Consensus was considered to be achieved if at least 75% of the panel (*n* = 23) agreed they had deliberately employed the CF care approach believing it was capable of influencing outcomes in patients with cLBP and none of the panel members rated the statement as ‘*Not Valid*’ or disagreed.

### Patient-practitioner relationship

#### Self-reported use, perceived as clinically valid, and self-confidence

During the first round (*n* = 39) the self-reported use of CF care approaches to develop the patient-practitioner relationship ranged from 76.9 to 100%. Similarly, CF care approaches which were perceived as potentially valid during the treatment of patients with cLBP ranged from 76.9 to 92.3%. Although 76.9% of the panel thought *applying different forms of touch* was perceived as a clinically valid care approach during the first round, there was insufficient consensus (73.9%) during the second round. The least frequently used diagnostic approach related to exploring the *meaning of the patient’s symptoms* (see Table 3, rank 16) with only 53.8% expressing self-confidence.

The self-reported use and perceptions regarding the acceptability of CF care approaches to improve the patient-practitioner relationship were generally higher than the panel’s self-confidence to apply them without undertaking further training. Less than 70% of the panel reported being confident about their non-verbal communication skills such as not interrupting the patient or using open body language. More importantly, less than 70% of the panellists were confident about using particular person-centred care approaches such as developing the therapeutic alliance, expressing genuine empathy, engaging in collaborative decision-making, or requesting the patient’s opinion. Table 3 below presents a summary of the first-round results.

**Table 3:** Panel’s self-reported use, perceived clinical validity, and confidence concerning the patient-practitioner relationship (Round 1; *n* = 39)

Rank	Sub-set	Statement	Self-reported use (%)	Valid (%)	Confidence (%)
*Patient-practitioner relationship (k = 17 statements)*					
2	Non-verbal behaviour	Being warm, confident, friendly, relaxed, and open during the appointment	100 (*n* = 39)	87.2 (*n* = 34)	79.5 (*n* = 31)
2	Non-verbal behaviour	Using eye contact, smiling, caring expressions of support and interest to convey empathy and compassion	100 (*n* = 39)	87.2 (*n* = 34)	76.9 (*n* = 30)
2*	Using specific diagnostic approach	Providing effective reassurance via clear and understandable explanations	100 (*n* = 39)	87.2 (*n* = 34)	71.8 (*n* = 28)
4.5	Using specific diagnostic approach	Examining the patient fully using appropriate therapeutic ‘hands on’ touch during the clinical examination	97.4 (*n* = 38)	87.2 (*n* = 34)	76.9 (*n* = 30)
4.5	Person-centred care approach	Ensuring the patient feels listened to and heard (e.g., active listening or noting their responses)	97.4 (*n* = 38)	87.2 (*n* = 34)	71.8 (*n* = 28)
6.5	Non-verbal behaviour	Not rushing or interrupting the patient; giving them time to tell their story	94.9 (*n* = 37)	89.7 (*n* = 35)	66.7 (*n* = 26)
6.5	Person-centred care approach	Engaging in collaborative decision-making with patients (e.g., mutually agreed and flexible goals)	94.9 (*n* = 37)	82.1 (*n* = 32)	66.7 (*n* = 26)
8*	Person-centred care approach	Providing treatment choices and encouraging patients to choose option(s) if they so wish	92.3 (*n* = 36)	87.2 (*n* = 34)	69.2 (*n* = 27)
9.5	Non-verbal behaviour	Using affirmative head nodding, forward leaning, open body postures/orientations	89.7 (*n* = 35)	84.6 (*n* = 33)	69.2 (*n* = 27)
9.5	Person-centred care approach	Promoting the patient’s sense of relatedness and partnership with you (i.e., therapeutic alliance)	89.7 (*n* = 35)	82.1 (*n* = 32)	64.1 (*n* = 25)
12	Person-centred care approach	Using verbal expressions of empathy, support, and language reciprocity (e.g., using the patient’s words/phrasing)	84.6 (*n* = 33)	92.3 (*n* = 36)	69.2 (*n* = 27)
12*	Person-centred care approach	Requesting the patient’s opinions and demonstrating you trust and respect them	84.6 (*n* = 33)	84.6 (*n* = 33)	64.1 (*n* = 25)
12	Person-centred care approach	Individualising the interaction style according to a patient’s preference (e.g., collaborative or authoritative)	84.6 (*n* = 33)	87.2 (*n* = 34)	59.0 (*n* = 23)
14*	Using specific diagnostic approach	Providing a detailed, definitive, and confident diagnosis	79.5 (*n* = 31)	79.5 (*n* = 31)	56.4 (*n* = 22)
16	Person-centred care approach	Adopting psychosocial talk or partnership statements (e.g., we, us, together)	76.9 (*n* = 30)	82.1 (*n* = 32)	66.7 (*n* = 26)
16	Non-verbal behaviour	Applying different forms of touch (e.g., assistive touch, touch to prepare the patient, touch to provide information, touch to reassure the patient)	76.9 (*n* = 30)	76.9 (*n* = 30)	66.7 (*n* = 26)
16	Using specific diagnostic approach	Asking questions about the meaning of the patient’s symptoms (i.e., what symptoms indicate to them)	76.9 (*n* = 30)	82.1 (*n* = 32)	53.8 (*n* = 21)

If two or more statements had equal percentages of self-reported use, then fractional ranks were computed by averaging the ordinal ranks to reflect ties. For example, three statements ranked combined “second” (i.e., (1 + 2 + 3)/3 = 2) and a rank of 4.5 indicates joint “fourth/fifth” (i.e., (4 + 5)/2 = 4.5) and so forth

*This statement was revised between the two Delphi rounds

#### Perceived influence: panel consensus

With regards to the patient-practitioner relationship, there was group-consensus for 18 of 19 statements included in the second round. Of these 18 statements, overall levels of agreement were high, ranging from 86.9 to 100%. For six statements, 100% of the panel agreed they had intentionally used non-verbal behaviours, person-centred care approaches, and cognitive reassurance believing it would influence clinical outcomes. Table 4 below presents a summary of these results. Notably, the only statement where panel consensus was below the 75% threshold (i.e., 73.9% agreement) involved *applying different forms of touch* (see Table 4, rank 19).

**Table 4:** Summary of panel’s agreement levels concerning the patient-practitioner relationship (Round 2; *n* = 23)

Rank	Sub-set	Statement	Mean (S.D.)	[95% CIs]	Agreement levels	Panel consensus	Percentage Disagree
*Patient-practitioner relationship (k = 19 statements)*							
1.5	Non-verbal behaviour	Using eye contact, smiling, caring expressions of support to convey empathy or compassion	4.74 (± 0.45)	[4.54, 4.93]	73.9% strongly agree 26.1% agree	Yes (100%)	0%
1.5	Using specific diagnostic approach	Providing a meaningful explanation of the patient’s LBP (i.e., cognitive reassurance) which is clear, understandable, and can be referred to after treatment	4.74 (± 0.45)	[4.54, 4.93]	73.9% strongly agree. 26.1% agree	Yes (100%)	0%
3.5	Person-centred care approach	Ensuring the patient feels listened to and heard (e.g., active listening or noting their responses)	4.70 (± 0.56)	[4.45, 4.94]	73.9% strongly agree 21.7% agree	Yes (95.7%)	4.3% (unsure)
3.5	Person-centred care approach	Individualising the interaction style according to a patient’s preference (e.g., collaborative, or authoritative)	4.70 (± 0.56)	[4.45, 4.94]	73.9% strongly agree 21.7% agree	Yes (95.7%)	4.3% (unsure)
5	Non-verbal behaviour	Being warm, friendly, and relaxed during the appointment	4.65 (± 0.49)	[4.44, 4.86]	65.2% strongly agree 34.8% agree	Yes (100%)	0%
6.5*	Person-centred care approach	Compassionately expressing your understanding of how LBP affects them (e.g., *'I understand how frustrating it is not to be able to walk your dog/go dancing/garden' *etc.)	4.61 (± 0.50)	[4.39, 4.82]	60.9% strongly agree 39.1% agree	Yes (100%)	0%
6.5	Person-centred care approach	Promoting the patient’s sense of relatedness and partnership with you (i.e., therapeutic alliance)	4.61 (± 0.58)	[4.36, 4.86]	65.2% strongly agree 30.4% agree	Yes (95.7%)	4.3% (unsure)
9*	Person-centred care approach	Confirming the patient not only heard but also understood the content of your communication	4.57 (± 0.51)	[4.35, 4.78]	56.5% strongly agree 43.5% Agree	Yes (100%)	0%
9	Non-verbal behaviour	Not rushing or interrupting the patient; giving them time to tell their story	4.57 (± 0.59)	[4.31, 4.82]	60.9% strongly agree 34.8% agree	Yes (95.7%)	4.3% (unsure)
9	Person-centred care approach	Engaging in collaborative decision-making together (e.g., mutually agreed, and flexible goals)	4.57 (± 0.66)	[4.28, 4.85]	65.2% strongly agree 26.1% agree	Yes (91.3%)	8.7% (unsure)
12.5	Person-centred care approach	Using verbal expressions of empathy, support, and language reciprocity (e.g., using the patient’s words)	4.52 (± 0.51)	[4.30, 4.74]	52.2% strongly agree 47.8% agree	Yes (100%)	0%
12.5	Using specific diagnostic approach	Examining the patient fully using appropriate therapeutic ‘hands on’ touch during the clinical examination	4.52 (± 0.59)	[4.27, 4.78]	56.5% strongly agree 39.1% agree	Yes (95.6%)	4.3% (unsure)
12.5	Non-verbal behaviour	Using affirmative head nodding, forward leaning, open body postures/orientations	4.52 (± 0.67)	[4.23, 4.81]	60.9% strongly agree 30.4% agree	Yes (91.3%)	8.7% (unsure)
12.5*	Person-centred care approach	Demonstrating you trust or respect the patient and their opinions	4.52 (± 0.67)	[4.23, 4.81]	60.9% strongly agree 30.4% agree	Yes (91.3%)	8.7% (unsure)
15	Using specific diagnostic approach	Asking questions about the meaning of the patient’s symptoms (i.e., what symptoms indicate to them). (*n* = 22)^a^	4.50 (± 0.60)	[4.24, 4.76]	54.5% strongly agree 40.9% agree	Yes (95.4%)	4.5% (unsure)
16	Using specific diagnostic approach	Providing a confident diagnosis (e.g., providing a diagram with simple explanations and/or notes)	4.43 (± 0.73)	[4.12, 4.75]	56.5% strongly agree 30.4% agree	Yes (86.9%)	13.0% (unsure)
17*	Using specific diagnostic approach	Explaining improvement(s) can be dynamic, and their condition/symptoms may change throughout treatment	4.39 (± 0.72)	[4.08, 4.70]	52.2% strongly agree 34.8% agree	Yes (87.0%)	13.0% (unsure)
18	Person-centred care approach	Adopting psychosocial talk or partnership statements (e.g., we, us, together)	4.22 (± 0.67)	[3.93, 4.51]	34.8% strongly agree 52.2% agree	Yes (87.0%)	13.0% (unsure)
19	Non-verbal behaviour	Applying different forms of touch (e.g., assistive touch, touch to prepare the patient, touch to provide information, touch to reassure the patient)	3.96 (± 0.83)	[3.60, 4.31]	26.1% strongly agree 47.8% agree	**No** (**73.9%**)	21.7% (unsure) 4.3% disagree

If two or more statements had equal means, then fractional ranks were computed by averaging the ordinal ranks to reflect ties. For example, rank 1.5 indicates joint “first/second” (i.e., (1 + 2)/2 = 1.5) and a rank of 3.5 indicates joint “third/fourth” (i.e., (3 + 4)/2 = 3.5) and so forth

*A new item suggested by a panel member during the first round

^a^Where *n* is < 23, the corresponding responses were excluded from the analysis if the response option ‘*Do not recall/use*’ was selected

### Patient’s beliefs and characteristics

#### Self-reported use, perceived as clinically valid, and self-confidence

The self-reported use of CF care approaches aiming to modify patient’s beliefs ranged from 51.3 to 100% and perceptions relating to the potential clinical validity during cLBP treatment ranged from 61.5 to 92.3%. The most commonly used CF care approaches which were also perceived as clinically acceptable included actively investigating the patient’s needs, feelings, preferences, and previous experiences, and supporting the patient in reframing negative memories (e.g., reinterpret an X-ray, explain radiological reports or GP letters). Notably, the panel’s self-reported use of approaches to modify patient’s individual beliefs was typically higher than their self-reported confidence.

The two most commonly used cognitive behavioural approaches involved reframing the patient’s prior LBP misconceptions and addressing inaccurate treatment beliefs whilst the least commonly used included helping a patient plan and monitor treatment success and empowering each patient to self-care. Less than 40% of the panel were confident to use these CF care approaches despite perceiving them as clinically acceptable. Contrastingly, more than 90% of the panel reported addressing unhelpful illness perceptions and fear-avoidance behaviours, although less than 60% expressed self-confidence*.* Table 5 below presents a summary of these results.

**Table 5:** Panel’s self-reported use, perceived clinical validity, and confidence addressing patient’s beliefs/characteristics (Round 1; *n* = 39)

Rank	Sub-set	Statement	Self-reported use (%)	Valid (%)	Confidence (%)
*Patient’s beliefs and characteristics (k = 23 statements)*					
1.5	Patient’s treatment history	Actively investigating patient’s needs, feelings, preferences, and previous experiences	100 (*n* = 39)	89.7 (*n* = 35)	74.4 (*n* = 29)
1.5	Patient’s treatment history	Supporting the patient in reframing negative memories (e.g., reinterpret an X-ray/scan or explain radiological reports/GP letters)	100 (*n* = 39)	89.7 (*n* = 35)	64.1 (*n* = 25)
3.5*	Cognitive behavioural approach	Reframing patient’s prior misconceptions about low back pain (e.g., *‘pain is not always a sign of physical tissue damage,’* ‘*your spine is flexible not fragile’*)	97.4 (*n* = 38)	87.2 (*n* = 34)	71.8 (*n* = 28)
3.5*	Patient’s treatment history	Taking note of inaccurate knowledge from previous treatment experiences (e.g., ‘*my spine is crumbling’* or ‘*my back is worn out’*)	97.4 (*n* = 38)	89.7 (*n* = 35)	69.2 (*n* = 27)
6.5	Cognitive behavioural approach	Reframing patient’s prior misconceptions about treatment (e.g., ‘*bed rest does not usually help patients recover faster but modified activity can*’)	94.9 (*n* = 37)	84.6 (*n* = 33)	71.8 (*n* = 28)
6.5	Reducing negative outcomes	Reinforcing a shift in patient’s negative thoughts to positive ones (e.g., outcomes to highlight progress)	94.9 (*n* = 37)	87.2 (*n* = 34)	59.0 (*n* = 23)
6.5	Cognitive behavioural approach	Clarifying maladaptive perceptions (e.g., catastrophising: ‘*My vertebrae are out of line. I stopped gardening, so I won’t end up in wheelchair*’)	94.9 (*n* = 37)	84.6 (*n* = 33)	59.0 (*n* = 23)
6.5*	Cognitive behavioural approach	Assisting in decreasing fear-avoidance and harm beliefs along with avoidant behaviours	94.9 (*n* = 37)	87.2 (*n* = 34)	59.0 (*n* = 23)
9	Creating positive outcomes	Communicating to patients an intervention is likely to be effective (e.g., ‘*this treatment usually works for most people with low back pain*’)	92.3 (*n* = 36)	89.7 (*n* = 35)	74.4 (*n* = 29)
11.5	Creating positive outcomes	Being optimistic during the consultation and regarding their dysfunction (e.g., ‘*I believe you will get back to your usual level of functioning again*’)	89.7 (*n* = 35)	89.7 (*n* = 35)	76.9 (*n* = 30)
11.5	Reducing negative outcomes	Allocating time for patients to ask about negative aspects of treatment	89.7 (*n* = 35)	89.7 (*n* = 35)	66.7 (*n* = 26)
11.5	Cognitive behavioural approach	Explaining the multi-dimensional nature (biopsychosocial aspects) of pain (i.e., beliefs, emotions, and behaviours (movement and lifestyle)) via suitable educational materials	89.7(*n* = 35)	87.2 (*n* = 34)	61.5 (*n* = 24)
11.5	Cognitive behavioural approach	Developing patient’s self-confidence in performing and persisting with a new behaviour to pursue a goal	89.7 (*n* = 35)	89.7 (*n* = 35)	51.3 (*n* = 20)
14	Reducing negative outcomes	Anticipating and helping reduce patient’s anxiety about the treatment/procedure	87.2 (*n* = 34)	92.3 (*n* = 36)	56.4 (*n* = 22)
15.5	Creating positive outcomes	Emphasising positive outcomes such as overall pain-reducing effects (e.g., ‘*manual or physical therapies are often as effective as painkillers*’)	82.1 (*n* = 32)	79.5 (*n* = 31)	66.7 (*n* = 26)
15.5*	Sociocultural context^a^	Displaying a balanced attitude to patient’s alternative or cultural beliefs if not harmful (e.g., acupuncture)	82.1 (*n* = 32)	82.1 (*n* = 32)	53.8 (*n* = 21)
17	Reducing negative outcomes	Avoiding negative phrases (e.g., ‘wear and tear,’ ‘damage’, ‘degeneration’, ‘ongoing’ instead of ‘chronic’ pain, ‘plan activities’ instead of ‘do exercise’)	79.5 (*n* = 31)	87.2 (*n* = 34)	56.4 (*n* = 22)
18	Reducing negative outcomes	Rephrasing negative information (e.g., during leg flexion test: ‘*this procedure may lead to a slight increase in pain’ rather say instead: ‘this procedure might be a bit uncomfortable but only temporarily’*)	76.9 (*n* = 30)	89.7 (*n* = 35)	59.0 (*n* = 23)
19.5*	Cognitive behavioural approach	Helping patients plan and monitor treatment success (e.g., SMART goals, motivational interviewing)	71.8 (*n* = 28)	87.2 (*n* = 34)	35.9 (*n* = 14)
19.5*	Cognitive behavioural approach	Empowering patients to self-care and anticipate barriers (e.g., reminders, implementation intentions, journal/logbook, NHS online self-care resources)	71.8 (*n* = 28)	89.7 (*n* = 35)	33.3 (*n* = 13)
21*	Sociocultural context^a^	Involving significant others and/or primary carers in treatment	69.2 (*n* = 27)	79.5 (*n* = 31)	46.2 (*n* = 18)
22.5*	Creating positive outcomes	Helping patients associate hands on techniques with positive outcomes using positive verbal instructions (e.g., ‘*I expect your pain will improve after this manipulation*’)	51.3 (*n* = 20)	61.5 (*n* = 24)	51.3 (*n* = 20)
22.5*	Reducing negative outcomes	Describing how (un)common side effects are numerically (e.g., 1 in 100 people)	51.3 (*n* = 20)	76.9 (*n* = 30)	38.5 (*n* = 15)

If two or more statements had equal percentages of self-reported use, then fractional ranks were computed by averaging the ordinal ranks to reflect ties. For example, rank 1.5 indicates joint “first/second” (i.e., (1 + 2)/2 = 1.5) and a rank of 3.5 indicates joint “third/fourth” (i.e., (3 + 4)/2 = 3.5) and so forth

*This statement was revised between the two Delphi rounds

^a^Statements relating to the socio-cultural context were not included in the second round

#### Perceived influence: panel consensus

There was group-consensus for 21 of 25 statements relating to patient’s beliefs and characteristics. For 21 statements, levels of agreement ranged from 82.6 to 100% indicating practitioners were actively using these CF care approaches to influence clinical outcomes. Of the five statements with 100% agreement, four related to the patient’s treatment history. Mean rankings suggest examining the patient’s treatment history by understanding their prior experiences and addressing misinformed beliefs were perceived as important CFs. Table 6 below presents a summary of these results.

Three statements where consensus was not achieved were new additions from the first round, even though agreement levels exceeded the 75% threshold (see Table 6), specifically, instilling hope (rank 16); explaining self-care involves managing stress (rank 18); and explaining why imaging is unnecessary (rank 23.5). Another statement was below the consensus threshold (73.9% agreement), namely, *emphasising positive outcomes such as overall pain-reducing effects* (see Table 6, rank 25), as 26.1% of the panellists were unsure whether this might influence patient outcomes.

**Table 6:** Summary of panel’s agreement levels concerning the patient’s beliefs/characteristics (Round 2; *n* = 23)

Rank	Sub-set	Statement	Mean (S.D.)	[95% CIs]	Agreement levels	Panel consensus	Percentage Disagree
*Patient’s beliefs and characteristics (k = 25 statements)*							
1	Patient’s treatment history	Reframing misinformed beliefs from previous healthcare experiences (e.g., *'my spine is crumbling', 'my spinal curve is abnormal', 'my back is worn out'*)	4.91 (± 0.29)	[4.79, 5.04]	91.3% Strongly Agree 8.7% Agree	Yes (100%)	0%
2	Patient’s treatment history	Actively investigating patient’s needs, feelings, preferences, and previous experiences	4.83 (± 0.39)	[4.66, 4.99]	82.6% Strongly Agree 17.4% Agree	Yes (100%)	0%
3	Patient’s treatment history	Supporting the patient in reframing negative memories (e.g., reinterpret an X-ray/scan or explain radiology reports/GP letters)	4.78 (± 0.42)	[4.60, 4.96]	78.3% Strongly Agree 21.7% Agree	Yes (100%)	0%
4.5	Reducing negative outcomes	Allocating time for patients to ask about negative aspects of treatment to address their concerns openly and honestly	4.70 (± 0.47)	[4.49, 4.90]	69.6% Strongly Agree 30.4% Agree	Yes (100%)	0%
4.5	Reducing negative outcomes	Anticipating and helping reduce patient’s anxiety about the treatment/procedure	4.70 (± 0.64)	[4.42, 4.97]	78.3% Strongly Agree 13.0% Agree	Yes (91.3%)	8.7% (unsure)
7*	Cognitive behavioural approach	Explaining routine activities, movement, or exercise can help 'rewire' perceived pain pathways (e.g., *some pain or discomfort is normal but is not a sign their LBP is "worsening")*	4.65 (± 0.57)	[4.40, 4.90]	69.6% Strongly Agree 26.1% Agree	Yes (95.7%)	4.3% (unsure)
7	Cognitive behavioural approach	Clarifying maladaptive perceptions (e.g., catastrophising: ‘*My vertebrae are out of line. I stopped gardening, so I won’t end up in a wheelchair’*)	4.65 (± 0.57)	[4.40, 4.90]	69.6% Strongly Agree 26.1% Agree	Yes (95.7%)	4.3% (unsure)
7	Cognitive behavioural approach	Developing patient’s self-confidence in performing or persisting with a new behaviour or goal	4.65 (± 0.65)	[4.37, 4.93]	73.9% Strongly Agree 17.4% Agree	Yes (91.3%)	8.7% (unsure)
10*	Patient’s treatment history	Exploring the patient’s current or pre-existing beliefs about the cause(s) of their LBP	4.61 (± 0.50)	[4.39, 4.82]	60.9% Strongly Agree 39.1% Agree	Yes (100%)	0%
10	Cognitive behavioural approach	Reframing patient’s prior misconceptions about treatment (e.g., ‘*bed rest does not usually help patients recover faster but modified activity can’*)	4.61 (± 0.58)	[4.36, 4.86]	65.2% Strongly Agree 30.4% Agree	Yes (95.7%)	4.3% (unsure)
10	Cognitive behavioural approach	Assisting in decreasing fear-avoidance and harm beliefs by recognising, confronting, and correcting them	4.61 (± 0.58)	[4.36, 4.86]	65.2% Strongly Agree 30.4% Agree	Yes (95.7%)	4.3% (unsure)
12.5	Cognitive behavioural approach	Helping patients plan and monitor treatment success (e.g., explain outcome measures; co-create short-term and long-term goals or target-driven stages of improvement)	4.57 (± 0.59)	[4.31, 4.82]	60.9% Strongly Agree 34.8% Agree	Yes (95.7%)	4.3% (unsure)
12.5	Creating positive outcomes	Communicating an intervention is likely to be effective using positive verbal instructions (e.g., *'I expect your pain will improve after treatment'*)	4.57 (± 0.59)	[4.31, 4.82]	60.9% Strongly Agree 34.8% Agree	Yes (95.7%)	4.3% (unsure)
14	Cognitive behavioural approach	Reframing patient’s prior misconceptions about their anatomy/physiology (e.g., *‘your spine is flexible not fragile’*)	4.52 (± 0.67)	[4.23, 4.81]	60.9% Strongly Agree 30.4% Agree	Yes (91.3%)	8.7% (unsure)
16	Reducing negative outcomes	Reinforcing a shift in patient’s negative thoughts to positive ones (e.g., monitor outcomes to highlight progress)	4.48 (± 0.59)	[4.22, 4.73]	52.2% Strongly Agree 43.5% Agree	Yes (95.7%)	4.3% (unsure)
16	Creating positive outcomes	Being optimistic during treatment by providing a prognosis (e.g., *'I believe you will recover and get back to your usual level of functioning'*)	4.48 (± 0.67)	[4.19, 4.77]	56.5% Strongly Agree 34.8% Agree	Yes (91.3%)	8.7% (unsure)
16*	Creating positive outcomes	Instilling genuine hope in patients regarding how their life can change for the better	4.48 (± 1.08)	[4.01,4.95]	65.2% Strongly Agree 30.4% Agree	**No (95.6%)**	**4.3% Not Valid**
18*	Reducing negative outcomes	Explaining that calming their stress response is a part of everyday self-care for physical pain and healing. (n = 22)^a^	4.45 (± 0.91)	[4.05, 4.86]	59.1% Strongly Agree 36.4% Agree	**No (95.5%)**	**4.5% Strongly Disagree**
20*	Cognitive behavioural approach	Explaining basic pain science (i.e., *perceived pain is not necessarily actual physical pain from nerve or tissue damage, but whilst very real, is more of a 'learned' response to prior experiences*)	4.43 (± 0.59)	[4.18, 4.69]	47.8% Strongly Agree 47.8% Agree	Yes (95.7%)	4.3% (unsure)
20	Cognitive behavioural approach	Explaining the multi-dimensional nature (biopsychosocial aspects) of pain (i.e., beliefs, emotions, and behaviours (movement and lifestyle)) via suitable educational materials	4.43 (± 0.79)	[4.09, 4.78]	60.9% Strongly Agree 21.7% Agree	Yes (82.6%)	17.4% (unsure)
20*	Reducing negative outcomes	Using simple, everyday analogies to alter patient's negative illness perceptions (e.g., ‘*rusty hinges often work well despite their appearance*’)	4.43 (± 0.79)	[4.09, 4.78]	60.9% Strongly Agree 21.7% Agree	Yes (82.6%)	17.4% (unsure)
22	Reducing negative outcomes	Avoiding negative phrases (e.g., ‘wear and tear’, ‘damage’, ‘degeneration’, 'abnormal')	4.35 (± 0.71)	[4.04, 4.66]	47.8% Strongly Agree 39.1% Agree	Yes (87.0%)	13.0% (unsure)
23.5	Reducing negative outcomes	Rephrasing negative information (e.g., leg flexion test: ‘*this procedure might be a bit uncomfortable but only temporarily*’)	4.26 (± 0.69)	[3.96, 4.56]	39.1% Strongly Agree 47.8% Agree	Yes (87.0%)	13.0% (unsure)
23.5*	Reducing negative outcomes	Explaining imaging is usually unnecessary because scans may not explain the extent of their pain and/or dysfunction	4.26 (± 0.96)	[3.84, 4.68]	47.8% Strongly Agree 39.1% Agree	**No (87.0%)**	8.7% (unsure) **4.3% Strongly Disagree**
25	Creating positive outcomes	Emphasising positive outcomes such as overall pain-reducing effects (e.g., ‘*manual or physical therapies are often as effective as painkillers*’)	4.22 (± 0.85)	[3.85, 4.59]	47.8% Strongly Agree 26.1% Agree	**No (73.9%)**	26.1% (unsure)

If two or more statements had equal means, then fractional ranks were computed by averaging the ordinal ranks to reflect ties. For example, a rank of 4.5 indicates joint “fourth/fifth” (i.e., (4 + 5)/2 = 4.5) and three statements ranked combined “seventh” (i.e., (6 + 7 + 8)/3 = 7) and so forth

*A new item suggested by a panel member during the first round

^a^Where *n* is < 23, the corresponding responses were excluded from the analysis if the response option ‘*Do not recall/use*’ was selected

### Practitioner’s beliefs and characteristics

#### Self-reported use, perceived as clinically valid, and perceived treatment effects

Self-reported use of CF care approaches relating to the practitioner’s own beliefs or characteristics ranged from 56.4 to 100%, whilst their perceptions regarding the potential clinical validity ranged from 53.8 to 89.7%. During the first round, the panel indicated whether they believed each CF care approach might enhance overall treatment effects instead of reporting their self-confidence. Table 7 below presents a summary of these results.

Notably, 100% of the panel reported adapting their mindset or attitude during treatment by remaining attentive and fully focused on patients and being genuine and honest to promote trustworthiness. More than 80% of the panel perceived these CF care approaches as clinically valid and thought they might enhance treatment effects (see Table 7, ranks 1.5). However, 59.0% of the panel reported wearing uniforms or formal clothing whilst only 53.8% perceived it as a clinically valid care approach (see Table 7, rank 6). Similarly, only 56.4% of the panel reported using indicators to tacitly display their expertise, although 66.7% thought these cues (e.g., qualifications) may enhance treatment effects (see Table 7, rank 7).

**Table 7:** Panel’s self-reported use, perceived clinical validity and effects of the practitioner’s beliefs/characteristics (Round 1; *n* = 39)

Rank	Sub-set	Statement	Self-reported use (%)	Valid (%)	Enhance Treatment (%)
*Practitioner’s beliefs and characteristics (k = 7 statements)*					
1.5	Mindset/attitude	Remaining attentive and fully focused on the patient throughout the appointment	100 (*n* = 39)	89.7 (*n* = 35)	84.6 (*n* = 33)
1.5	Mindset/attitude	Being genuine and honest to instil a sense of trustworthiness and authenticity	100 (*n* = 39)	87.2 (*n* = 34)	82.1 (*n* = 32)
3*	Mindset/attitude	Displaying self-confidence without appearing arrogant or dismissive	97.4 (*n* = 38)	84.6 (*n* = 33)	79.5 (*n* = 31)
4	Expertise/credibility	Clearly communicating your expectations (i.e., what you anticipate will occur) whilst administering care	94.9 (*n* = 37)	84.6 (*n* = 33)	74.4 (*n* = 29)
5*	Expertise/credibility	Prescribing or administering treatments you believe and expect to be effective	92.3 (*n* = 36)	82.1 (*n* = 32)	76.9 (*n* = 30)
6*	Expertise/credibility	Wearing a laboratory coat/medical apparel or tailored/formal clothing to symbolise professionalism	59.0 (*n* = 23)	53.8 (*n* = 21)	59.0 (*n* = 23)
7*	Expertise/credibility	Using indicators of expertise/high status (e.g., health qualifications, professional memberships) in offices or correspondence	56.4 (*n* = 22)	59.0 (*n* = 23)	66.7 (*n* = 26)

If two or more statements had equal percentages of self-reported use, then fractional ranks were computed by averaging the ordinal ranks to reflect ties. For example, rank 1.5 indicates joint “first/second” (i.e., (1 + 2)/2 = 1.5)

*This statement was revised between the two Delphi rounds

#### Perceived influence: panel consensus

There was group-consensus for 10 of 11 statements related to the practitioner’s beliefs and characteristics during the second round, with overall levels of agreement ranging from 91.3 to 100% suggesting practitioners were actively adapting their mindset or attitude and demonstrating their expertise believing it could influence clinical outcomes. There were three statements where 100% of the panel agreed that their mindset or attitude could enhance cLBP treatment (see Table 8, ranks 1–3)*.*

However, panel consensus was not met regarding the use of indicators (e.g., qualifications, professional memberships) in clinics, online, or via correspondence (71.4% agreement). Practitioners preferred to demonstrate their expertise by clearly communicating their expectations, only administering treatments they expected to be effective, and demonstrating professionalism through their general appearance (e.g., being clean, tidy, and presentable) rather than wearing a medical uniform. A summary of these results is presented in Table 8 below.

**Table 8:** Summary of panel’s agreement levels concerning the practitioner’s beliefs/characteristics (Round 2; *n* = 23)

Rank	Sub-set	Statement	Mean (S.D.)	[95% CIs]	Agreement levels	Panel consensus	Percentage Disagree
*3) Practitioner’s beliefs and characteristics (k = 11 statements)*							
1	Mindset/attitude	Remaining attentive and fully focused on the patient throughout the appointment	4.87 (± 0.34)	[4.72, 5.02]	87.0% Strongly Agree 13.0% Agree	Yes (100%)	0%
2	Mindset/attitude	Being genuine and honest to instil a sense of trustworthiness and authenticity	4.83 (± 0.39)	[4.66, 4.99]	82.6% Strongly Agree 17.4% Agree	Yes (100%)	0%
3*	Mindset/attitude	Displaying a professional and caring (not only "curing") attitude	4.78 (± 0.42)	[4.60, 4.96]	78.3% Strongly Agree 21.7% Agree	Yes (100%)	0%
4.5*	Mindset/attitude	Being calm and compassionate throughout the appointment	4.70 (± 0.56)	[4.45, 4.94]	73.9% Strongly Agree 21.7% Agree	Yes (95.7%)	4.3% (unsure)
4.5	Expertise/credibility	Clearly communicating your expectations (i.e., what you anticipate will occur) whilst administering care	4.70 (± 0.64)	[4.42, 4.97]	78.3% Strongly Agree 13.0% Agree	Yes (91.3%)	8.7% (unsure)
6.5	Expertise/credibility	Administering treatments you expect to be effective	4.61 (± 0.58)	[4.36, 4.86]	65.2% Strongly Agree 30.4% Agree	Yes (95.7%)	4.3% (unsure)
6.5	Mindset/attitude	Displaying self-confidence without appearing dismissive	4.61 (± 0.58)	[4.36, 4.86]	65.2% Strongly Agree 30.4% Agree	Yes (95.7%)	4.3% (unsure)
8*	Mindset/attitude	Creating a caring atmosphere (e.g., appear to have all the time in the world; ensure each patient feels like a priority)	4.52 (± 0.59)	[4.27, 4.78]	56.5% Strongly Agree 39.1% Agree	Yes (95.7%)	4.3% (unsure)
9.5	Expertise/credibility	Demonstrating professionalism through your general appearance (i.e., being clean, tidy, smart, and presentable)	4.48 (± 0.59)	[4.22, 4.73]	52.2% Strongly Agree 43.5% Agree	Yes (95.7%)	4.3% (unsure)
9.5*	Mindset/attitude	Actively build rapport with each patient (e.g., discuss common interests/hobbies; enquire about their lives)	4.48 (± 0.67)	[4.19, 4.77]	56.5% Strongly Agree 34.8% Agree	Yes (91.3%)	8.7% (unsure)
11	Expertise/credibility	Using indicators to display your expertise or credibility (e.g., qualifications, insurance, professional memberships) in reception/office, website, or correspondence. (*n* = 21)^a^	4.00 (± 0.89)	[3.59, 4.41]	33.3% Strongly Agree 38.1% Agree	**No (71.4%)**	23.8% (unsure) **4.8% Disagree**

If two or more statements had equal means, then fractional ranks were computed by averaging the ordinal ranks to reflect ties. For example, a rank of 4.5 indicates joint “fourth/fifth” (i.e., (4 + 5)/2 = 4.5) and so forth

*A new item suggested by a panel member during the first round

^a^Where *n* is < 23, the corresponding responses were excluded from the analysis if the response option ‘*Do not recall/use*’ was selected

### Treatment characteristics

#### Self-reported use, perceived as clinically valid, and perceived treatment effects

Using treatment characteristics ranged from 30.8 to 89.7% whilst perceptions regarding the potential clinical validity ranged from 53.8 to 89.7%. More than 80% of panellists reported encouraging patients to try activity reinforcement strategies and engaging in treatment/exercise with an optimistic mindset. Although continuity of care was commonly used and considered to be a clinically valid care approach during the first round (87.2%), two panellists disagreed during the second round, despite 87.0% believing it might influence patient outcomes.

Only 53.8% of the panel thought increasing the frequency/duration of appointments to provide extra time or attention was a clinically valid care approach, but 64.1% thought it might enhance treatment effects. Providing alternative feedback or encouraging engagement with other patients (see Table 9, ranks 7 and 8 respectively) experiencing positive results were not commonly used nor viewed as clinically valid care approaches*.* Table 9 below presents a summary of these results.

**Table 9:** Panel’s self-reported use, perceived clinical validity, and effects of treatment characteristics (Round 1; *n* = 39)

Rank	Sub-set	Statement	Self-reported use (%)	Valid (%)	Enhance Treatment (%)
*Treatment characteristics (k = 8 statements)*					
1	Appointment features	Ensuring the patient is cared for by the same practitioner/therapist (i.e., continuity of care)	89.7 (*n* = 35)	87.2 (*n* = 34)	79.5 (*n* = 31)
2.5	Treatment advice or options	Overtly encouraging patients to engage in therapy/exercise with an optimistic mindset to try establish positive associations with pain relief	84.6 (*n* = 33)	87.2 (*n* = 34)	76.9 (*n* = 30)
2.5	Treatment advice or options	Encouraging patients to find suitable incentives/reinforcement strategies to increase daily activity (e.g., personalised activities, exercise partners)	84.6 (*n* = 33)	89.7 (*n* = 35)	69.2 (*n* = 27)
4*	Treatment advice or options	To show and tell the patient that as a therapy is applied it helps (e.g., ‘*I am applying pressure here because it helps…*’)	66.7 (*n* = 26)	61.5 (*n* = 24)	66.7 (*n* = 26)
5*	Appointment features	Verbalising future treatment plans by stating the number of appointments and/or follow-ups (e.g., ‘*I will treat you every second week for 30 min’*)	61.5 (*n* = 24)	66.7 (*n* = 26)	64.1 (*n* = 25)
6	Appointment features	Increasing the frequency and/or duration of appointments (i.e., provide extra time/attention)	59.0 (*n* = 23)	53.8 (*n* = 21)	64.1 (*n* = 25)
7	Alternative feedback	Administering treatments along with visual feedback (e.g., using mirrors during exercises)	41.0 (*n* = 16)	71.8 (*n* = 28)	61.5 (*n* = 24)
8*	Alternative feedback	Enabling patients to engage with other patients undergoing treatment with positive results (e.g., group exercise classes, sharing success stories/testimonials, informally in the waiting area)	30.8 (*n* = 12)	59.0 (*n* = 23)	51.3 (*n* = 20)

If two or more statements had equal percentages of self-reported use, then fractional ranks were computed by averaging the ordinal ranks to reflect ties. For example, rank 2.5 indicates joint “second/third” (i.e., (2 + 3)/2 = 2.5)

*This statement was revised between the two Delphi rounds

#### Perceived influence: panel consensus

There was group-consensus for six of 12 statements relating to treatment characteristics during the second round with agreement levels ranging between 82.6 and 100%. CF care approaches which were perceived to be influential included using reinforcement strategies to increase daily activity, explaining treatment advice in line with patient's expectations, encouraging an optimistic mindset during therapy/exercise, providing self-management materials, demonstrating functional changes following treatment, and providing a patient with clear milestones to demonstrate progress. A summary of these results is presented in Table 10 below.

There was insufficient panel consensus for the remaining six statements; four were below the 75% threshold, whilst another two exceeded it, but panellists rated the statement as ‘*Not Valid*’ and/or expressed disagreement. Two involved modifying appointment features such as ensuring continuity of care and increasing the frequency or duration of appointments. Using verbal or visual feedback (e.g., sharing positive patient stories, or mirrors during exercises) were also not considered to be beneficial nor was explaining the difference between a clinical examination and treatment.

**Table 10:** Summary of panel’s agreement levels concerning the treatment characteristics (Round 2; *n* = 23)

Rank	Sub-set	Statement	Mean (S.D.)	[95% CIs]	Agreement levels	Panel consensus	Percentage Disagree
*Treatment characteristics (k = 12 statements)*							
1	Treatment advice or options	Encouraging patients to find suitable incentives/reinforcement strategies to increase daily activity (e.g., personalised activities, exercise partners)	4.52 (± 0.51)	[4.30, 4.74]	52.2% Strongly Agree 47.8% Agree	Yes (100%)	0%
2*	Treatment advice or options	Explaining your treatment advice in line with the patient's treatment expectations	4.48 (± 0.67)	[4.19, 4.77]	56.5% Strongly Agree 34.8% Agree	Yes (91.3%)	8.7% (unsure)
3	Treatment advice or options	Overtly encouraging patients to engage in therapy/exercise with an optimistic mindset to try establish positive associations with pain relief. (*n* = 22)^a^	4.45 (± 0.74)	[4.13, 4.78]	59.1% Strongly Agree 27.3% Agree	Yes (86.4%)	13.6% (unsure)
4*	Treatment advice or options	Demonstrating whether functional change has occurred immediately after treatment (e.g., pain, range of motion, or strength)	4.39 (± 0.67)	[4.11, 4.68]	47.8% Strongly Agree 43.5% Agree	Yes (91.3%)	8.7% (unsure)
5*	Treatment advice or options	Providing self-management materials (e.g., videos, rehabilitation booklets) or email/telephone support to promote a patient's engagement in physical activities. (*n* = 22)^a^	4.32 (± 0.65)	[4.03, 4.60]	40.9% Strongly Agree 50.0% Agree	Yes (90.9%)	9.1% (unsure)
6.5	Alternative feedback	Providing patients with clear milestones or signposting to indicate their progression through the treatment programme	4.22 (± 0.74)	[3.90, 4.54]	39.1% Strongly Agree 43.5% Agree	Yes (82.6%)	17.4% (unsure)
6.5	Appointment features	Ensuring the patient is cared for by the same practitioner/therapist (i.e., continuity of care)	4.22 (± 1.20)	[3.70, 4.74]	52.2% Strongly Agree 34.8% Agree	**No (87.0%)**	4.3% (unsure)
							**4.3% Disagree**
							**4.3% Not Valid**
8	Alternative feedback	Displaying feedback from other patients to provide reassurance (i.e., testimonials displayed on TV in waiting area, or online via website). (*n* = 17)^a^	3.88 (± 0.99)	[3.37, 4.39]	29.4% Strongly Agree 41.2% Agree	**No (70.6%)**	17.6% (unsure)
							**11.8**%** Disagree**
9*	Alternative feedback	Sharing positive stories of other (anonymous) patients with similar problems or goals. (*n* = 22)^a^	3.86 (± 1.08)	[3.38, 4.34]	22.7% Strongly Agree 54.5% Agree	**No (77.3%)**	18.2% (unsure)
							**4.5% Not Valid**
10	Alternative feedback	Administering treatments along with visual feedback (e.g., using mirrors during exercises). (*n* = 20)^a^	3.80 (± 1.06)	[3.31, 4.29]	30.0% Strongly Agree 35.0% Agree	**No (65.0%)**	20.0% (unsure)
							**15.0% Disagree**
11	Appointment features	Increasing the frequency and/or duration of appointments (i.e., provide extra time/attention). (*n* = 22)^a^	3.64 (± 1.43)	[3.00, 4.27]	36.4% Strongly Agree 22.7% Agree	**No (59.1%)**	22.7% (unsure)
							**9.1% Disagree**
							**4.5% Strongly Disagree**
							**4.5% Not Valid**
12*	Treatment advice or options	Clearly explaining the difference between a clinical examination and treatment. (*n* = 21)^a^	3.62 (± 0.97)	[3.18, 4.06]	23.8% Strongly Agree 23.8% Agree	**No (47.6%)**	42.9% (unsure)
							**9.5% Disagree**

*A new item suggested by a panel member during the first round

^a^Where *n* is < 23, the corresponding responses were excluded from the analysis if the response option ‘*Do not recall/use*’ was selected

### Treatment environment/setting

#### Self-reported use, perceived as clinically valid, and perceived treatment effects

Using CF care approaches to enhance the treatment environment ranged from 46.2 to 92.3% whilst the perceptions of their potential clinical validity ranged from 56.4 to 82.1%. Ensuring adequate privacy for patients was most commonly used, whereas positive distractors (e.g., soothing music, nice aromas) were used less frequently. Overall, less than 60% of the panellists thought altering the décor or layout was likely to enhance the overall treatment effects except for providing privacy, natural lighting, and ensuring a comfortable temperature. Table 11 below presents a summary of these results.

**Table 11:** Panel’s self-reported use, perceived clinical validity and effects of the treatment environment (Round 1; *n* = 39)

Rank	Sub-set	Statement	Self-reported use (%)	Valid (%)	Enhance Treatment (%)
*Treatment environment/setting (k = 9 statements)*					
1	Interior design/layout	Ensuring treatment facilities have privacy provisions (e.g., private changing area and treatment room, curtains/blinds on windows)	92.3 (*n* = 36)	82.1 (*n* = 32)	61.5 (*n* = 24)
2*	Interior design/layout	Considering seating provisions in treatment office (e.g., relative position to desk, additional chairs for carer)	87.2 (*n* = 34)	79.5 (*n* = 31)	59.0 (*n* = 23)
3.5	Setting’s décor	Waiting areas and treatment facilities are uncluttered and tidy	84.6 (*n* = 33)	71.8 (*n* = 28)	59.0 (*n* = 23)
3.5*	Setting’s décor	Decorating the waiting area with cheerful ornamentation (e.g., healthy indoor plants, leisure reading materials, comfortable cushions)	84.6 (*n* = 33)	71.8 (*n* = 28)	59.0 (*n* = 23)
5	Interior design/layout	Ensuring facilities have ample natural light or windows, and are suitably heated/ventilated (i.e., comfortable temperature)	79.5 (*n* = 31)	79.5 (*n* = 31)	69.2 (*n* = 27)
6	Setting’s décor	Providing visual indicators or cues to signify it is a medical setting (e.g., model of spine, patient information brochures, medicalised décor)	71.8 (*n* = 28)	64.1 (*n* = 25)	53.8 (*n* = 21)
7*	Interior design/layout	Considering seating provisions in the waiting areas (e.g., quantity, varying chair sizes, general arrangement)	64.1 (*n* = 25)	74.4 (*n* = 29)	59.0 (*n* = 23)
8*	Setting’s décor	Using nature artworks that include green vegetation, flowers, or water may help to reduce anxiety	48.7 (*n* = 19)	59.0 (*n* = 23)	59.0 (*n* = 23)
9*	Setting’s décor	Combining positive distractors such as soft or soothing music, nice aromas, hot or cold beverages	46.2 (*n* = 18)	56.4 (*n* = 22)	59.0 (*n* = 23)

If two or more statements had equal percentages of self-reported use, then fractional ranks were computed by averaging the ordinal ranks to reflect ties. For example, rank 3.5 indicates joint “third/fourth” (i.e., (3 + 4)/2 = 3.5)

*This statement was revised between the two Delphi rounds

#### Perceived influence: panel consensus

There was only group-consensus for three of seven statements relating to the treatment environment. Of these, agreement levels ranged from 87.0 to 91.3%. All three related to the interior design including providing adequate privacy, ample natural lighting, a comfortable temperature, and ensuring clinic facilities are tidy*.* Contrastingly, there was insufficient consensus regarding the clinic’s décor (36.4–69.6%). Despite exceeding the 75% threshold, one panellist disagreed that rearranging furniture or seating in treatment rooms influenced patient outcomes. These results are summarised in Table 12 below.

**Table 12:** Summary of panel’s agreement levels concerning the treatment environment or setting (Round 2; *n* = 23)

Rank	Sub-set	Statement	Mean (S.D.)	[95% CIs]	Agreement levels	Panel consensus	Percentage Disagree
*Treatment environment/setting (k = 7 statements)*							
1	Interior design/layout	Ensuring treatment facilities have privacy provisions (e.g., private changing area and treatment room, curtains/blinds on windows)	4.52 (± 0.67)	[4.23, 4.81]	60.9% Strongly Agree 30.4% Agree	Yes (91.3%)	8.7% (unsure)
2	Interior design/layout	Rearranging the furniture or seating provisions in the treatment office (e.g., relative position to desk, additional chairs for carer)	4.35 (± 0.89)	[3.97, 4.73]	56.5% Strongly Agree 26.1% Agree	**No (82.6%)**	13.0% (unsure) **4.3% Disagree**
3	Setting’s décor	Ensuring waiting areas and treatment facilities are uncluttered and tidy	4.22 (± 0.67)	[3.93, 4.51]	34.8% Strongly Agree 52.2% Agree	Yes (87.0%)	13.0% (unsure)
4	Interior design/layout	Ensuring treatment facilities have ample natural light or windows, and are suitably heated/ventilated (i.e., comfortable temperature)	4.13 (± 0.55)	[3.89, 4.37]	21.7% Strongly Agree 69.6% Agree	Yes (91.3%)	8.7% (unsure)
5	Setting’s décor	Creating a positive ambience or atmosphere (e.g., flowers, plants, interesting magazines, friendly staff, relaxing background music, warm lighting)	3.87 (± 1.22)	[3.34, 4.40]	34.8% Strongly Agree 34.8% Agree	**No (69.6%)**	21.7% (unsure) **4.3% Disagree** **4.3**%** Not Valid**
6	Setting’s décor	Providing visual indicators or cues to signify it is a medical setting (e.g., model of spine, patient information brochures, medicalised décor)	3.61 (± 1.27)	[3.06, 4.16]	30.4% Strongly Agree 21.7% Agree	**No (52.1%)**	34.8% (unsure) **8.7% Disagree** **4.3**%** Not Valid**
7	Setting’s décor	Using nature artworks that include green vegetation, flowers, or water features. (*n* = 22)^a^	3.36 (± 1.14)	[2.86, 3.87]	18.2% Strongly Agree 18.2% Agree	**No (36.4%)**	54.5% (unsure) **4.5% Disagree** **4.5% Not Valid**

^a^Where *n* is < 23, the corresponding responses were excluded from the analysis if the response option ‘*Do not recall/use*’ was selected

### Perceived importance of CFs

The panel rated the patient-practitioner relationship as the most important CF whilst the treatment environment/setting was perceived as the least important CF during the treatment of patients with cLBP. Summary statistics for each of the main CF domains are presented in Table 13 below.

**Table 13:** Summary statistics rating the perceived importance of main CF domains (Round 2; *n* = 23)

Rank	Main CF domain	Mean (S.D.)	95% Confidence Interval	Median; Interquartile Range (Min–Max)
1	Patient-practitioner relationship	6.17 (± 0.65)	5.89–6.46	6.00; 1 (5–7)
2	Patient’s beliefs and characteristics	6.09 (± 0.73)	5.77–6.40	6.00; 1 (5–7)
3	Practitioner’s beliefs and characteristics	5.78 (± 0.74)	5.46–6.10	6.00; 1 (4–7)
4	Treatment characteristics	5.48 (± 1.08)	5.01–5.95	6.00; 1 (2–7)
5	Treatment environment/setting	4.91 (± 1.00)	4.48–5.34	5.00; 2 (3–7)

Question: *On a scale ranging from 1 (not at all important) to 7 (extremely important), based on your experience and beliefs, please rate the importance of each contextual factor to the patient's treatment during the healthcare encounter*

Response options: 1 – Not at all important; 2 – Low importance; 3 – Slightly important; 4 – Neutral; 5 – Moderately important; 6 – Very important; 7 – Extremely important

**Fig. 6:**
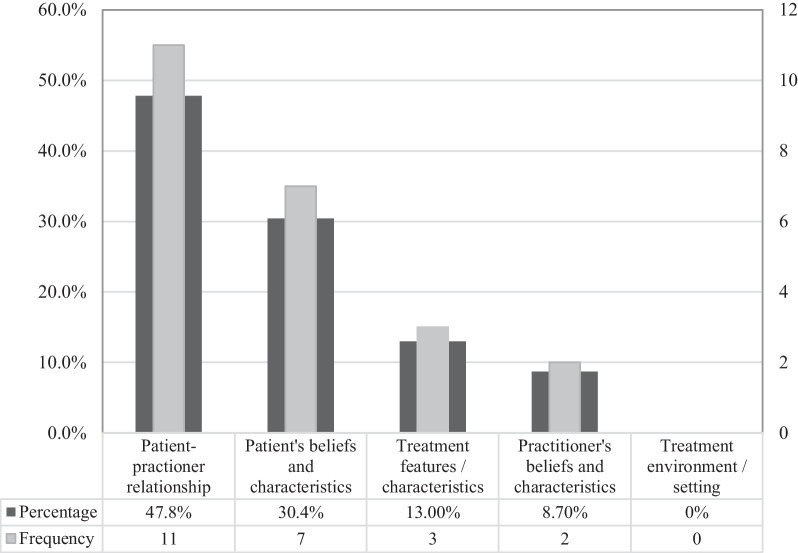
Panel’s perception regarding the most important CF domain during cLBP treatment

**Fig. 7:**
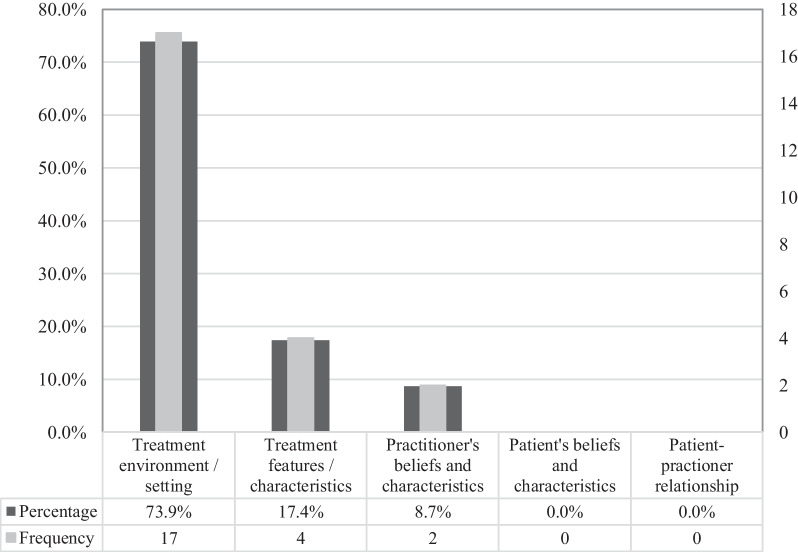
Panel’s perception regarding the least important CF domain during cLBP treatment

Additionally, the panel were asked to select one of the main CF domains which they perceived as being the most and least important during the treatment of patients with cLBP. Similar to the results presented in Table 13, Fig. 6 below indicates that nearly half the panel selected the patient-practitioner relationship (47.8%; *n* = 11) as the most important CF, followed by the patient’s beliefs and characteristics (30.4%; *n* = 7). Contrastingly, Fig. 7 below demonstrates the majority of the panel rated the treatment environment/setting (73.9%; *n* = 17) as the least important CF during cLBP treatment.

## Discussion

Recently, a range of CFs within therapeutic encounters have been highlighted as potentially influencing placebo analgesia in clinical practice for patients with MSK conditions and non-malignant pain [12–15]. These CF care approaches have not been widely evaluated amongst MSK practitioners to determine whether they are perceived as clinically acceptable and/or whether they are being deliberately harnessed during everyday clinical practice. Clinicians’ views and use of CFs is limited [2, 18], particularly in relation to specific MSK conditions. Accordingly, this Delphi study aimed to examine the extent to which a UK panel of MSK practitioners perceived CFs as acceptable modulators of outcomes for patients with cLBP and their use in clinical practice to determine if there was group consensus.

This Delphi study found three useful insights. Firstly, the UK panel of MSK practitioners perceived that all five CF domains (i.e., the patient-practitioner relationship, the patient’s and the practitioner’s beliefs/characteristics, the treatment characteristics, and environment [4]) were capable of influencing cLBP outcomes. Secondly, practitioners reported a lack of confidence in applying some of these CF care approaches, and these findings suggest potential training opportunities which could assist MSK practitioners in better adopting CFs aimed at supporting a positive therapeutic encounter. Lastly, the panel’s collective views indicated that the patient-practitioner relationship was perceived as the most important CF during cLBP treatment.

### Agreement with the five main CF domains

The UK panel demonstrated a high degree of consensus regarding the perceived influence, perceived clinical validity or acceptability and intentional use of person-centred communication, non-verbal behaviours, and diagnostic practices such as effective reassurance to enhance the *patient-practitioner relationship*. This is consistent with findings from a national survey of Italian MTs (*n* = 558) as the most beneficial CFs included developing an empathic therapeutic alliance and using a person-centred approach [2]. Similarly, the therapeutic relationship was rated as the most important CF in a national survey of Italian physiotherapists (*n* = 699) where key practices included adopting a person-centred approach, active listening, paraphrasing, and metaphors to facilitate improved patient understanding [18]. Essential CF care approaches for developing the patient-practitioner relationship include expressing empathy, warmth, friendliness, and authentic interest or involvement [5]. Purposeful body language to demonstrate active listening, genuine concern, and responsiveness to the patient can also strengthen the relationship [5].

Three beneficial inter-related care approaches in acute care settings included therapeutic listening, person-centredness, and responding to the patient’s emotions and unmet needs [55]. These approaches were associated with improvements in quality of life, anxiety and depression, treatment adherence, and patient satisfaction. Contrastingly negative interactions were linked to psychological distress as patients felt invalidated or dehumanised [55]. Likewise, key factors influencing the patient-practitioner relationship during MSK treatment include the practitioner’s interpersonal and communication skills; practical training and expertise; ability to provide patient education; person-centred and individualised care; along with time and flexible appointments [56]. Notably, there was a lack of consensus by the UK panel regarding the influence of different forms of touch (e.g., to assist, reassure or provide information) which differs from the Italian MTs [2] and may indicate cultural differences concerning the perceived effects of touch during MSK treatment.

The UK panel also exhibited a high degree of consensus regarding *patient’s beliefs and characteristics,* perceiving these CFs as acceptable modulators of clinical outcomes during cLBP management. The patient’s history and prior experiences were consistently viewed as influential CFs, along with attempting to reduce a patient’s anxiety about their treatment and discussing any concerns. Anticipatory anxiety activates cholecystokinin which facilitates pain transmission and is implicated in the nocebo response [19]. Accordingly, the UK panel may be helping to reduce anticipatory anxiety and potentially preventing negative outcomes by understanding each patient’s prior experiences along with actively managing their anxiety and addressing their specific concerns. Likewise, the Italian MTs thought the patient’s expectations, preferences, and previous experiences had potentially beneficial effects and often used these approaches on a weekly or daily basis [2]. The Italian physiotherapists rated the patient’s characteristics and beliefs as the second most important CF whilst noting the most useful approaches related to stimulating positive expectations and taking the patient’s expectations into account [18]. In our Delphi study there was insufficient panel consensus regarding the role of imaging, stress-management, instilling hope in recovery, or emphasising the pain-reducing effects of manual/physical therapies. Explaining severe injury or illness has been ruled out combined with a thorough physical examination may help reassure patients scans are unnecessary [57]. Furthermore, person-centred education to address misinformed pain-related beliefs and verbal suggestions to influence symptom change expectations may augment conservative treatment in patients with cLBP [54].

The UK panel displayed a high degree of consensus regarding *practitioner’s beliefs and characteristics* as CFs capable of influencing clinical outcomes. In our Delphi study, being attentive, kind, calm, compassionate, genuine, honest, creating a caring atmosphere, and ensuring every patient feels prioritised were consistently used and perceived as influential approaches to build trust. However, there was insufficient panel consensus regarding the use of indicators to display their expertise. Preferred ways to demonstrate professionalism included clearly communicating their expectations, and wearing clean, smart clothing rather than a uniform. Uniforms were also not viewed as important by the Italian MTs and physiotherapists [2, 18] but were often worn by MTs in the private sector or hospitals [2]. The Italian MTs believed their professional reputation might have some beneficial effects but did not frequently use it [2], whilst the Italian physiotherapists rated communication strategies as the most important way to demonstrate their professionalism, followed by their reputation, and hygiene/cleanliness [18]. In a recent systematic review, higher levels of clinician/experimenter confidence, competence, professionalism, as well as positive body language (e.g., smiling, tone of voice, eye-contact) modulated pain [58]. This highlights the importance of MSK practitioners being mindful of how patients might perceive their body language and professional attitudes, as subtle cues can influence pain [1, 15, 58].

The UK panel reached consensus for half the statements concerning the *treatment characteristics* including using reinforcement strategies to increase daily activity, providing self-management materials, encouraging an optimistic mindset during therapy, explaining treatment advice in line with a patient’s expectations, and demonstrating functional changes following treatment. Important needs of patients include a clear understanding of their LBP [59], consistent, comprehensible, and individualised information relating to their prognosis, treatment options, and self-management tools, which consider their work and healthcare concerns [60]. Notably, there was insufficient panel consensus regarding the use of visual feedback (e.g., mirrors), altering appointment features, ensuring continuity of care, or sharing positive stories of other (anonymous) patients to provide reassurance. It is possible that MSK practitioners are unaware of the role of social or observational learning mechanisms associated with placebo analgesia [1, 3, 15]. Italian MTs reported using mirrors and physical contact to inform, assist, prepare, and take care of the patient on a daily basis [2] which differs from the UK panel and might indicate another cultural difference. The treatment characteristics were rated the third most important CF by the Italian physiotherapists [18], although comparable statements (e.g., one-to-one versus group sessions, and price) were not included in our Delphi study.

The *treatment environment* was perceived as the least important CF overall, and group-consensus was only achieved for three statements relating to the interior design, namely, adequate privacy, uncluttered treatment facilities, and a comfortable environment. The UK panel’s views are comparable to the Italian MTs and physiotherapists as both focused on a comfortable environment [2, 18]. A comfortable setting was viewed as more beneficial for patients than the architecture (windows, skylights) or the use of decorations, ornaments, and colours amongst Italian MTs [2]. Using relaxing music, soft lighting and creating a comfortable treatment setting may provide an opportunity to manage negative emotions such as fear or anxiety, which are common in patients with MSK pain [15, 50–52]. Rehn and Schuster [61] emphasise how appropriate design elements evoke expectations which can promote healing and support treatment by influencing patients’ experiences and health behaviour. Consequently, there may be a missed opportunity to improve patient outcomes by leveraging additional features of the treatment environment.

### Lack of confidence in applying CFs

Despite recognising the patient-practitioner relationship as the most important CF, the UK panel were not entirely confident in applying a range of person-centred care approaches. Furthermore, these MSK practitioners were not altogether confident handling patients’ negative emotional states, explaining the multi-dimensional nature of pain, using cognitive-behavioural approaches to challenge unhelpful beliefs/behaviours, cultivating self-efficacy, or promoting self-management strategies. This is important because it helps identify skills gaps which may support the optimal use of CFs during cLBP rehabilitation.

A growing body of evidence suggests emotional and cognitive factors influence pain processing, pain-related distress, and coping responses in patients with cLBP [57, 62]. Accordingly, a key recommendation of this Delphi study is MSK practitioners require further training to enhance their proficiency and confidence in applying essential psychosocial skills to address the complex needs of patients with cLBP. For instance, educational interventions to assist MSK practitioners in changing patients’ unhelpful illness beliefs may serve to augment the treatment of pain-related disability [54, 63]. Another example may include targeted interventions to address MSK practitioners misinformed or erroneous beliefs (e.g., use of imaging scans for LBP management/diagnosis) [63]. Similarly, adopting a framework to promote person-centredness in MSK practice may help to cultivate and enhance the therapeutic relationship (see [64] for applied clinical principles). Moreover, different training formats (e.g., face-to-face, and online) should be used to inform clinicians about placebo/nocebo effects [37]. Supporting practitioners’ skills development and confidence through bespoke short courses, workshops/seminars, which include practical exercises and activities, may be beneficial. Additionally, co-creating such interventions with both patients and practitioners may help ensure common challenges encountered during LBP rehabilitation are incorporated.

### Perceived importance of CFs

The UK panel’s collective ratings may indicate some of the main CF domains were perceived as more important during the treatment of patients with cLBP. The *patient-practitioner relationship* was generally perceived as the most important CF, followed by the *patient’s beliefs and characteristics,* with higher levels of panel consensus for these respective CF domains. The *practitioner’s beliefs and characteristics* were rated as the third most important, followed by the *treatment characteristics,* whereas the Italian physiotherapists rated them vice versa [18]. Both the UK panel and the Italian physiotherapists [18] perceived the *treatment environment* as the least important CF overall. However, these questionnaires were not identical, which may explain these differences to some extent. Notably, in our Delphi study, there was limited variability between these main CF domains. It may therefore be useful for future studies to consider using a larger sample of MSK practitioners to determine if there is sufficient evidence to indicate a hierarchy of importance regarding the use of CFs during clinical practice. Additionally, whether there is a hierarchy of importance that is reflected by clinical outcomes remains to be studied.

Future research might consider developing a standardised and validated questionnaire to investigate practitioners’ awareness, attitudes towards, and use of CFs during clinical practice. Greco and colleagues [65] have developed the Healing Encounters and Attitudes Lists (HEAL) for patients, but an equivalent version is not available for practitioners. It is therefore challenging to make direct comparisons across regions and professions because there is a lack of uniformity on how these broad CF domains have been operationalised and measured.

### Strengths and limitations

Strengths of the current study was the use of piloting to refine the statements included in the Delphi to ensure reasonable face and content validity. Additionally, statements were extracted from a range of sources which may have reduced researcher bias, but also provides an extensive array of CF care approaches which may be beneficial in clinical practice. The self-reported use of CFs during the management of patients with cLBP was relatively high. It is possible the UK panellists may have (inadvertently) responded in a socially desirable manner and it is unclear how frequently or consistently these approaches were applied. Furthermore, panel members self-selected to participate in this Delphi study based on their interest in the topic of CFs and their expertise as MSK practitioners. Accordingly, it is likely that self-selection/recruitment bias occurred, which may mean the panel’s perceptions may not represent the views of other MSK practitioners who are less familiar with, or less interested in the topic of CFs, or those working within public healthcare settings (NHS). For this reason, it would be worthwhile to test these findings using a larger sample size along with aiming to reduce selection bias in future. Further limitations include: the response options differing between rounds, as this may have affected the overall methodological rigour; the time lag between iterations, arising from the impact of Covid-19 during data collection, which may have affected the overall response rates; and that a study protocol was not pre-registered, which is recommended for future research.

Lastly, since a conservative approach was used to define panel consensus, the authors acknowledge this may have skewed some of the results (i.e., where agreement levels exceeded 75% but panel consensus was not achieved as a result of dissenting opinion(s)). The authors recognise percentage cut-off points are somewhat arbitrary and may impact the overall interpretation of the data. However, including cases of minority dissenting views does not appear to have substantively altered the conclusions. A conservative approach was taken since those expressing dissent might give further information regarding other MSK practitioners’ views which may provide an indication of skills/knowledge gaps or  identify potential barriers for the future implementation of CFs during routine clinical practice.

## Conclusion

This Delphi study provides initial insights regarding a panel of UK MSK practitioners’ attitudes towards the influence, use, and relative importance of CFs during cLBP treatment. All five CF domains were perceived as capable of influencing patient outcomes, with the *patient-practitioner relationship* being perceived as the most important CF during routine clinical practice. Various skills gaps were highlighted where supplementary training may support MSK practitioners’ capacity to address their patients’ complex cognitive and emotional needs. Increasing practitioners’ knowledge of CFs may help them to optimally harness these therapeutic effects and potentially improve patients’ outcomes during cLBP rehabilitation.

## Supplementary Information

**Additional file 1**: **Table S1**. Synopsis of new statements included in second round survey. **Table S2**. Summary of amendments to statements between rounds. Copy of Delphi Survey – Round 1 (DS–R1). Copy of Delphi Survey – Round 2 (DS–R2).

## Data Availability

The dataset generated and/or analysed during the current study are not publicly available yet since it will be published in Bournemouth University’s online research data repository (BORDaR) following the completion of the dissertation. It is available from the corresponding author on reasonable request and with the permission of Bournemouth University via a data sharing agreement.

## Abbreviations

CFs: Contextual factors
CI: Confidence Interval
MSK: Musculoskeletal
MTs: Manual therapists
LBP: Low back pain
cLBP: Chronic low back pain
UK: United Kingdom
CREDES: Conducting and REporting DElphi studies
NHS: National Health Service
HEAL: Healing Encounters and Attitudes Lists
