# Supplementary material for: Musculoskeletal practitioners’ perceptions of contextual factors that may influence chronic low back pain outcomes: a modified Delphi study
Source: Chiropr Man Therap. 2023 Apr 5;31:12. doi: 10.1186/s12998-023-00482-4 (PMC10075502; doi:10.1186/s12998-023-00482-4)
Supplement: Supplementary file 1 — Additional file 1: Table S1. Synopsis of new statements included in second round survey. Table S2. Summary of amendments to statements between rounds. Copy of Delphi Survey – Round 1 (DS–R1). Copy of Delphi Survey – Round 2 (DS–R2). [file 12998_2023_482_MOESM1_ESM.pdf]

List of Supplementary Information

Table S1. Synopsis of new statements included in second round survey

Table S2. Summary of amendments to statements between rounds

Copy of Delphi Survey – Round 1 **(DS–R1)**

Copy of Delphi Survey – Round 2 **(DS–R2)**

Table S1. Synopsis of new statements included in second round survey

**Practitioner's Beliefs and Characteristics**			
**Sub-Category**	**No.**	**Round-2: New Statement**	**Originated from Panel Suggestion(s)**
Mindset / attitude	Q8.4	Being calm and compassionate throughout the appointment.	*"Calm, compassionate, confidence - this comes with experience"*
Mindset / attitude	Q8.5	Displaying a professional and caring (not only "curing") attitude.	*"Displaying a "caring" (not only "curing") but professional attitude to patient's"*
Mindset / attitude	Q8.6	Creating a caring atmosphere (e.g., *appear to have all the time in the world; ensure each patient feels like a priority*).	– *"Attentive, kind, caring and appearing to have all the time in the world! It is important to make patients feel valued and cared for, and as if they are the only patient that you are seeing that day"*<br>– *"Being kind and having empathy"*
Mindset / attitude	Q8.7	Actively build rapport with each patient (e.g., *discuss common interests / hobbies; enquire about their lives*).	– *"Having similar hobbies as a Pt - I see many pts who ride horses and I ride - they believe I understand their ssx better because it affects them while riding and they believe I have a better understanding of that than someone who doesn't ride horses."*<br>– *"Remembering specifics of that pts life, e.g., did your daughter pass GCSE?"*
**Patient's Beliefs and Characteristics**			
Patient's treatment history	Q9.4	Exploring the patient's current or pre-existing beliefs about the cause(s) of their LBP.	– *"Exploring a patient's current beliefs about the cause of their problem"*
Creating ***positive*** *outcomes*	Q9.8	Instilling genuine hope in patients regarding how their life can change for the better.	*"Give patients genuine hope for how their life can change for the better - and what life improving activities they could return to"*
Reducing ***negative*** *outcomes*	Q10.3	Using simple, everyday analogies to alter patient's negative illness perceptions (e.g., *rusty hinges often work well despite their appearance*).	*"Using simple, everyday analogies to help explanations e.g., 'rusty' hinges working well, taking your car for a gentle drive every day to keep it moving, rather than driving it once a month by which time it'll have seized up - aids understanding"*
Reducing ***negative*** *outcomes*	Q10.7	Explaining that calming their stress response is a part of everyday self-care for physical pain and healing.	"*That calming the stress reaction is a part of their self-care for physical pain and healing."*
Reducing ***negative*** *outcomes*	Q10.8	Explaining imaging is usually unnecessary because scans may not explain the extent of their pain and/or dysfunction.	*"Explaining that imaging is not often necessary - and that what is seen on imaging doesn't necessarily equate to the amount of pain or 'damage' - a bit like a rusty hinge looks pretty rubbish, but actually functions pretty well"*
Cognitive behavioural approach	Q11.4	Explaining basic pain science (i.e., perceived pain is not necessarily actual physical pain from nerve or tissue damage, but whilst very real, is more of a 'learned' response to prior experiences).	*"Explaining basic 'pain science' i.e., explaining that perceived pain is not necessarily actual physical pain from e.g., tissue damage, but whilst very real, is more of a 'learned' behaviour/response to a relatively benign, non-noxious stimulus"*
Cognitive behavioural approach	Q11.5	Explaining routine activities, movement, or exercise can help 'rewire' perceived pain pathways (e.g., *some pain or discomfort is normal but is not a sign their LBP is "worsening"*).	– *"Explaining that normal activities, movement, exercise etc can help 'rewire' perceived pain pathways and help 'normalise' pain"*<br>– *"Explaining that (some) pain during attempts at activity and ADL does not indicate tissue "damage" or "worsening" of the complaint (LBP)".*

Table S1 continued. Synopsis of new statements included in second round survey

Sub-Category	No.	Round-2: New Statement	Originated from Panel Suggestion(s)
**Patient-Practitioner Relationship**			
Using specific diagnostic approach	Q12.7	Explaining improvement(s) can be dynamic, and their condition / symptoms may change throughout treatment.	*"We don't always get the diagnosis right first time. Also improvement can be dynamic, and the diagnosis change."*
Person-centred care approach	Q13.2	Compassionately expressing your understanding of how LBP affects them (e.g., *'I understand how frustrating it is not to be able to walk your dog / go dancing / garden' etc*).	– *"Displaying empathy and understanding for how its life is affected by their condition. Such as "I understand how frustrating it is to not be able to play with your grandkids/go dancing anymore/garden etc"*<br>– *"Validation (normal for someone in their situation to feel the emotions they have)"*
Person-centred care approach	Q13.9	Confirming the patient not only heard but also understood the content of your communication.	"*Making sure that, after communicating with the patient, the patient has not only heard but understood the content of the communication"*
**Treatment Characteristics**			
Treatment advice or options	Q14.3	Clearly explaining the difference between a clinical examination and treatment.	– *"Explain clearly what is examination and what is treatment"*<br>– *"Possibility to have a phone call with me (on demand) to answer questions prior to initial appointment"*<br>– *"A lot of information about what to expect from the initial appointment on website and provided over the phone"*
Treatment advice or options	Q14.4	Demonstrating whether functional change has occurred immediately after treatment (e.g., *pain, range of motion, or strength*).	– *"Document changes in function by regular testing patient's ROM, pain and strength"*<br>– *"Patients will believe results more than explanations, and we should have the humility to accept this."*<br>– *"Show patients if functional change has occurred immediately after treatment such as pain, ROM, strength"*<br>– *"Carrying out post treatment examination (as appropriate)"*
Treatment advice or options	Q14.5	Explaining your treatment advice in line with the patient's treatment expectations.	– *"Explaining the advice in line with the treatment expectations"*<br>– *"Framing explanations based on their functional limitations and functional goals"*
Alternative feedback	Q14.8	Providing patients with clear milestones or signposting to indicate their progression through the treatment programme.	– *"Have a fully laid out plan with various stages of improvement driven by targets."*<br>– *"Reminding Pt how far they have come and noting changes e.g., you got on the table MUCH better than last week"*<br>– *"Providing patients with specific rehabilitation sessions to give them a sense of progressing during treatment programme and demonstrate exercises in secure environment"*

Table S2. Summary of amendments to statements between rounds

**Practitioner's Beliefs and Characteristics**		
**Original Statement (R1)**	**Rephrased Statement (R2)**	***Panel Input* / (Notes)**
Prescribing or administering treatments you believe and expect to be effective	Administering treatments you expect to be effective.	Pilot participant – *"ambiguous / double-barrelled"*
Displaying self-confidence without appearing arrogant or dismissive	Displaying self-confidence without appearing dismissive.	"*Whilst patients need to be involved in their care, they also expect confidence from their care-giver"*
Using indicators of expertise / high status (e.g., *health qualifications, professional memberships*) in offices or correspondence	Using indicators to display your expertise or credibility (e.g., *qualifications, insurance, professional memberships*) in reception / office, website, or correspondence.	"*Have certificates of qualification, insurance, registration on display in reception area, website etc, . It provides reassurance and credibility. Be subtle, not blatant bragging!"*
Wearing a laboratory coat / medical apparel or tailored / formal clothing to symbolise professionalism	Demonstrating professionalism through your general appearance (i.e., being clean, tidy, smart, and presentable).	– *"No uniform"*<br>– *"Professionalism - in manner, dress etc. Always need to be clean, tidy and presentable. I think a white coat is a barrier as it can be unapproachable, but smart, professional dress in important"*
Taking note of inaccurate knowledge from previous treatment experiences (e.g., *'my spine is crumbling'* or '*my back is worn out'*)	Reframing misinformed beliefs from previous healthcare experiences (e.g., *'my spine is crumbling', 'my spinal curve is abnormal', 'my back is worn out'*).	"*Reframing misinformation they may have been told by other practitioners without creating cognitive dissonance. i.e., many patients are told by other HCPs that they have an "abnormal spinal curve" without being given further info or reassurance*."
**Patient's Beliefs and Characteristics**		
Helping patients plan and monitor treatment success (e.g., SMART goals, motivational interviewing)	Helping patients plan and monitor treatment success (e.g., *explain outcome measures; co-create short-term and long-term goals or target-driven stages of improvement*).	Pilot participant – *"Using goal setting for short and longer term, i.e., what they want to do once things are a bit better and then a lot better - using these as benchmarks through course of treatment"*
Communicating to patients an intervention is likely to be effective (e.g., '*this treatment usually works for most people with low back pain'*)	Communicating an intervention is likely to be effective using positive verbal instructions (e.g., *'I expect your pain will improve after treatment'*).	(Two original statements were combined to reduce repetition / redundant items)
Helping patients associate hands on techniques with positive outcomes using positive verbal instructions (e.g., *'I expect your pain will improve after this manipulation'*)		
Being optimistic during the consultation and regarding their dysfunction (e.g., '*I believe you will get back to your usual level of functioning again'*)	Being optimistic during treatment by providing a prognosis (e.g., *'I believe you will recover and get back to your usual level of functioning'*).	*"Treatment must also include prognosis"*

Table S2 continued. Summary of amendments to statements between rounds

**Patient's Beliefs and Characteristics continued**		
**Original Statement (R1)**	**Rephrased Statement (R2)**	***Panel Input* / (Notes)**
Rephrasing negative information (e.g., during leg flexion test: *'this procedure may lead to a slight increase in pain'* rather say instead: *'this procedure might be a bit uncomfortable but only temporarily'*)	Rephrasing negative information (e.g., leg flexion test: '*this procedure might be a bit uncomfortable but only temporarily*').	(Statement simplified / refined)
Reframing patient's prior misconceptions about low back pain (e.g., *'pain is not always a sign of physical tissue damage'*, *'your spine is flexible not fragile'*)	Reframing patient's prior misconceptions about their anatomy / physiology (e.g., '*your spine is flexible not fragile*').	(Statement simplified / refined)
Assisting in decreasing fear-avoidance and harm beliefs along with avoidant behaviours	Assisting in decreasing fear-avoidance and harm beliefs by recognising, confronting, and correcting them.	*"Recognising, and confronting and correcting pre-existing fear-avoidance beliefs and behaviours"*
Avoiding negative phrases (e.g., '*wear and tear*', '*damage'*, *'degeneration',* 'ongoing' instead of *'chronic'* pain, 'plan activities' instead of '*do exercise'*)	Avoiding negative phrases (e.g., 'wear and tear', 'damage', 'degeneration', 'abnormal').	(Statement simplified / refined)
Requesting the patient's opinions and demonstrating you trust and respect them	Demonstrating you trust or respect the patient and their opinions.	(Statement simplified / refined)
Allocating time for patients to ask about negative aspects of treatment	Allocating time for patients to ask about negative aspects of treatment to address their concerns openly and honestly.	*"Honesty - if a patient asks if it is going to hurt, or cause side effects, you MUST be honest with them, allowing time for them to relay concerns and then allay them openly and honestly."*
Displaying a balanced attitude to patient's alternative or cultural beliefs if not harmful (e.g., acupuncture).	Deleted.	(Beyond original scope of CFs)
Involving significant others and/or primary carers in treatment.	Deleted.	(Beyond original scope of CFs)
Describing how (un)common side effects are numerically (e.g., 1 in 100 people).	Deleted.	(Reduce number of statements in this CF domain; relatively less important)
**Patient-Practitioner Relationship**		
Being warm, confident, friendly, relaxed, and open during the appointment	Being warm, friendly, and relaxed during the appointment.	Pilot participant – *"ambiguous / double-barrelled*"
Using eye contact, smiling, caring expressions of support and interest to convey empathy and compassion	Using eye contact, smiling, caring expressions of support to convey empathy or compassion.	(Statement simplified / refined)
Providing effective reassurance via clear and understandable explanations	Providing a meaningful explanation of the patient's LBP (i.e., cognitive reassurance) which is clear, understandable, and can be referred to after treatment.	– "*Explanation of the patient's particular problem in such a way that they can understand their condition"*<br>– *"Providing cognitive reassurance i.e., providing meaningful information that patients can use when they are outside the treatment room"*

Table S2 continued. Summary of amendments to statements between rounds

**Treatment Characteristics**		
**Original Statement (R1)**	**Rephrased Statement (R2)**	***Panel Input* / (Notes)**
Enabling patients to engage with other patients undergoing treatment with positive results (e.g., *group exercise classes, sharing success stories / testimonials, informally in the waiting area*)	Displaying feedback from other patients to provide reassurance (i.e., *testimonials displayed on TV in waiting area, or online via website*).	*"Displaying feedback from other patients to provide reassurance i.e., testimonials displayed on TV in waiting area."*
Enabling patients to engage with other patients undergoing treatment with positive results (e.g., *group exercise classes, sharing success stories / testimonials, informally in the waiting area*)	Sharing positive stories of other (anonymous) patients with similar problems or goals.	Pilot participant – *"Using (anon) positive stories of people I have seen with similar problem/goal"*
Empowering patients to self-care and anticipate barriers (e.g., *reminders, implementation intentions, journal / logbook, NHS online self-care resources*)	Providing self-management materials (e.g., *videos, rehabilitation booklets*) or email / telephone support to promote a patient's engagement in physical activities	– "*Empowering patient by demonstrating self-treatment activities that reduce the need for practitioner intervention. Otherwise, the patient becomes dependent on the treatment approach"*<br>– *"Patient education is important. In the long run they will be looking after themselves, and need to have the information to take responsibility for their health."*<br>– *"Providing email by support to increase the likelihood they do exercises or engage in physical activities"*<br>– *"Providing videos and other material by email"*
Verbalising future treatment plans by stating the number of appointments and/or follow-ups (e.g., *'I will treat you every second week for 30 minutes*')	Providing patients with clear milestones or signposting to indicate their progression through the treatment programme.	– *"Have a fully laid out plan with various stages of improvement driven by targets."*<br>– *"Reminding pt how far they have come and noting changes e.g., you got on the table MUCH better than last week"*<br>– *"Providing patients with specific rehabilitation sessions to give them a sense of progressing during treatment programme and demonstrate exercises in secure environment"*
Providing a detailed, definitive, and confident diagnosis	Providing a confident diagnosis (e.g., providing a diagram with simple explanations and/or notes).	– *"I don't believe a specific diagnosis is possible in low back pain /orthopaedics"*<br>– *"Providing the patient with a (pre-printed with a diagram) sheet where notes and explanations in relation to their particular complaint have been written (in basic language)"*<br>– *"I do offer what I the assessment has shown and what we might be able to conclude"*
To show and tell the patient that as a therapy is applied it helps (e.g., '*I am applying pressure here because it helps…*').	Deleted.	(Reduce number of statements in this CF domain; similar new item included)

Table S2 continued. Summary of amendments to statements between rounds

**Treatment Environment / Setting**		
Decorating the waiting area with cheerful ornamentation (e.g., *healthy indoor plants, leisure reading materials, comfortable cushions*)	Creating a positive ambience or atmosphere (e.g., flowers, plants, interesting magazines, friendly staff, relaxing background music, warm lighting)	*"Flowers, plants, interesting magazines, friendly staff, relaxing background music (classical works well), warm, bright light."*<br><br>(Two original statements were combined to reduce repetition / redundant items)
Combining positive distractors such as soft or soothing music, nice aromas, hot or cold beverages		
Considering seating provisions in the waiting areas (e.g., quantity, varying chair sizes, general arrangement).	Deleted.	(Similar statement included regarding seating provisions in treatment office.)
Using nature artworks that include green vegetation, flowers, or water may help to reduce anxiety.	Using nature artworks that include green vegetation, flowers, or water features.	Pilot participant – *" remove 'may help to reduce anxiety' it may be leading"*<br><br>*"Flowers, plants, interesting magazines, friendly staff, relaxing background music (classical works well), warm, bright light."*

Copy of Delphi Survey – Round 1 (DS-R1)

<u>Page 1: Welcome and Introduction</u>

Welcome to this Delphi-study regarding the perceived influence of contextual factors during treatment of chronic low back pain patients.

Intended For:

This survey is designed for:

- Qualified **manual and physical practitioners** (i.e., physiotherapists, chiropractors, and osteopaths);
- With three **(3) or more years' experience** in providing care for patients with **chronic low back pain**;
- Currently practicing in the **United Kingdom** (England, Scotland, Wales, and Northern Ireland).

Introduction:

- Manual and physical therapists use a variety of tools to achieve shared therapeutic goals such as improving patient's pain, physical functioning, and self-perceived health.
- Modifying contextual factors, including psychosocial aspects of care, are a promising supplementary approach to usual care for pain, which can potentially induce pain modulation and influence clinical outcomes via the following domains:

1. **patient's characteristics and beliefs** (e.g., preferences, previous experiences, gender, age);
2. **practitioner's characteristics and beliefs** (e.g., reputation, appearance, beliefs and behaviours);
3. **the patient-practitioner relationship** (e.g., communication, trust, patient-centred approach);
4. **the treatment features or characteristics** (e.g., clear diagnosis, overt therapy, therapeutic touch);
5. **the physical environment / setting** (e.g., environment, interior design).

- Contextual factors are therapeutic cues which may be essential for the perception and interpretation of care, which can be interpreted positively or negatively, but may dually affect symptom perception, experience, and meaning.

Next Steps:

- The next page contains a **Participant Information Sheet** to help you make an informed decision.
- If you are willing to participate in the first survey, please **select the Consent Statement checkbox** at the bottom of the next page and then **click 'next'** to begin the survey.
- The questionnaire is expected to take between **15 and 20 minutes** to complete.
- Survey **responses are collected over encrypted SSL (TLS) connections** to ensure information is transmitted securely.
- You will be able to provide your email address at the end of the survey if you are interested in future participation in the second panel-round.

Page 2: Participant Information Sheet and Consent

Ethics ID: 28052 **Version 1.0** **30.10.2019**

Participant Information Sheet

The title of the research project
Perceived influence of contextual factors during chronic low back pain treatment: a Delphi-consensus survey

What is the purpose of the research / questionnaire?
This study aims to explore your perceptions of the influence of five main types of contextual factors (that include psychosocial aspects of care) during the management of chronic low back pain patients. Since this study is a Delphi-consensus survey, there will be two consecutive online survey rounds consisting of short questionnaires. You will be given the opportunity to take part in both surveys, but participation in either one is voluntary.
The purpose of the first round is: to request your expert knowledge on care approaches you regularly use for patients with chronic low back pain; and to provide your opinion of and evaluate care approaches extracted from the literature and incorporated into this questionnaire. This Delphi-study forms part of a broader research project which is being conducted in order to obtain a PhD qualification from Bournemouth University, in partnership with AECC University College.

Why have I been chosen?
You are being asked to participate as we would like to understand physiotherapists', chiropractors', and osteopaths' views of contextual factors. We are seeking qualified practitioners, working in the UK, with three (3) or more years' experience providing regular care for patients with chronic low back pain. Expert opinion is required because of the limited research evidence currently available.

Do I have to take part?
It is entirely up to you to decide whether or not to take part. If you do decide to participate, you will have access to this online information sheet to read. If you do choose to proceed, beginning the online survey will mean that you agree to take part. You can withdraw from participating during the online survey at any time and without giving a reason. If you decide to withdraw, you can simply close the browser page, and this will remove any data collected about you from the study. Please note that once you have completed and submitted your survey responses, we are unable to remove your anonymised responses from the study. However, if you choose to provide your email address for the follow-up survey (second round), then your responses will be identifiable, and can then be removed. As we are unable to remove anonymised responses this can only be done prior to your email address being confidentially destroyed.

How long will the questionnaire / online survey take to complete?
Taking part will involve completing an online survey at a time convenient to you. The questionnaire is expected to take between 15 and 20 minutes to complete. You may also opt to *'finish later'* and either email yourself a copy of the link or leave the browser window open and continue at a later time.

What are the advantages and possible disadvantages or risks of taking part?
Whilst there are no immediate benefits for those people participating in the project, it is hoped that findings from this study will help improve outcomes for patients with chronic low back pain. The information collected will provide valuable insights into practitioners' views of contextual factors for clinical application during conservative care. There are no anticipated disadvantages of taking part in the survey, other than a small amount of time required to complete the voluntary questionnaires.

What type of information will be sought from me and why is the collection of this information relevant for achieving the research project's objectives?
The survey has five sections relating to each of the contextual factors. The main questions are closed-ended (i.e., checkbox response options) and relate to your beliefs about contextual factors and their role in clinical practice. There are also optional open-ended questions for you to provide suggestions based on your knowledge and expertise. The final section relates to basic demographic information (e.g. age, gender, practitioner type, years of clinical experience) which will be useful for categorisation and statistical analysis. This data will be used to develop the second round of the Delphi-consensus survey.

At the end of the survey, you will be given the option of providing your email address if you wish to express interest in participating in the follow-up survey. This is the only personally identifiable information requested and it will be kept confidential. An expression of interest simply indicates you might be willing to take part in the second round.

Use of my information
Participation in this study is on the basis of consent: you do not have to complete the survey, and you can change your mind at any point before submitting the survey responses. Once we receive your survey response, your personal information is processed in compliance with the data protection legislation. We will use your data on the basis that it is necessary for the conduct of research, which is an activity in the public interest.

Bournemouth University (BU) is a Data Controller of your information which means that we are responsible for looking after your information and using it appropriately. BU's Research Participant Privacy Notice sets out more information about how we fulfil our responsibilities as a data controller and about your rights as an individual under the data protection legislation. We ask you to read this Notice so that you can fully understand the basis on which we will process your information.
Once you have submitted your survey response it may not be possible for us to remove it from the study analysis, as this might affect our ability to complete the research appropriately, or the accuracy and reliability of the research findings.

Security and access controls
BU will hold the information we collect about you on a secure password protected BU network. Except where it has been anonymised, your personal information will only be accessed and used by appropriate, authorised individuals and when this is necessary for the purposes of the research or another purpose identified in the Privacy Notice. This may include giving access to BU staff or others responsible for monitoring and/or audit of the study, who need to ensure that the research is complying with applicable regulations.

Sharing and further use of your personal information
The information collected about you may be used in an anonymous form to support other research projects in the future and access to it in this format will not be restricted. It will not be possible for you to be identified from this data. Anonymised data will be added to BU's Data Repository (a central location where data is stored) and will be publicly available. You will not be able to be identified in the PhD thesis nor any external reports / publications about the research.

Retention of your data
Once the second round of email invitations / follow-ups are sent out, your email address will be confidentially destroyed. All other personal data collected for the purposes of this study will be held for three (3) years after the award of the degree. Although published research outputs are anonymised, we need to retain underlying data collected for the study in a non-anonymised form for a certain period to enable the research to be audited and/or to enable the research findings to be verified.

Contact for further information
If you would like to contact the main researcher (Bronwyn Sherriff) to raise any concerns or request further information, please direct your enquiries to: bsherriff@bournemouth.ac.uk
Alternatively, you are welcome to contact any one of my PhD supervisors:
Prof. Carol Clark: cclark@bournemouth.ac.uk
Prof. David Newell: dnewell@aecc.ac.uk
Dr Clare Killingback: c.killingback@hull.ac.uk

In case of complaints
Any concerns which have not been answered by the researchers should be directed to Professor Vanora Hundley, Faculty of Health and Social Sciences, Bournemouth University by emailing researchgovernance@bournemouth.ac.uk

This study has been approved by the University Research Ethics Committee (UREC) of Bournemouth University **(Ethics I.D. 28052)**

*1.***Consent to Participate** *Required*

<u>Page 3: Example Question</u>

For each statement, you will be able to ***select / tick all applicable column(s)*** if:
a) You believe the statement reflects a potentially valid care approach;
b) It is an approach/technique you currently use as part of your everyday practice;
c) It is an approach/technique you feel confident to use without further training / experience.

Example Question

For each statement, you will be able to ***select / tick all applicable column(s)*** if:

a) You believe the statement reflects a potentially valid care approach;
b) It is an approach/technique you currently use as part of your everyday practice;
c) It is an approach/technique you feel confident to use without further training / experience;

For Example:

What is your opinion of the following statements?

	**Please tick applicable box(es)**			
	**a) I think it is a valid approach**	**b) I use this approach in practice**	**c) I am confident to use without training**	**Not applicable**
**e.g., Switching treatment approaches if a patient expresses prior negative experiences**	✓	✓	✓	
**e.g., Ensuring treatment areas and equipment are clean**		✓	✓	
**e.g., Showing signs of being in a hurry (e.g., talking quickly)**				✓
**e.g., Matching the practitioner and patient according to characteristics (e.g., gender, culture, home language)**			✓	

Page 4: (1) Patient's Beliefs and Characteristics

Please ***select / tick all applicable column(s)*** if:
a) You believe the statement below reflects a potentially valid care approach;
b) It is an approach/technique you currently use as part of your everyday practice;
c) It is an approach/technique you feel confident to use without further training / experience.

---

*2.*What is your opinion of the following aspects of the *patient's treatment history*?

	**Please tick applicable box(es)** *Required*			
	a) I think it is a valid approach	b) I use this approach in practice	c) I am confident to use without training	Not Applicable
1. Actively investigating patient's needs, feelings, preferences, and previous experiences.	☐	☐	☐	☐
2. Supporting the patient in reframing negative memories (e.g., reinterpret an x-ray / scan or explain radiological reports / GP letters).	☐	☐	☐	☐
3. Taking note of inaccurate knowledge from previous treatment experiences (e.g., 'my spine is crumbling' or 'my back is worn out').	☐	☐	☐	☐

*3.*What is your opinion of attempting to create *positive outcomes* via the following approaches?

	**Please tick applicable box(es)** *Required*			
	a) I think it is a valid approach	b) I use this approach in practice	c) I am confident to use without training	Not Applicable
1. Communicating to patients an intervention is likely to be effective (e.g., 'this treatment usually works for most people with low back pain').	☐	☐	☐	☐
2. Emphasising positive outcomes such as overall pain-reducing effects (e.g., 'manual or physical therapies are often as effective as painkillers').	☐	☐	☐	☐
3. Being optimistic during the consultation and regarding their dysfunction (e.g., 'I believe you will get back to your usual level of functioning again').	☐	☐	☐	☐
4. Helping patients associate hands on techniques with positive outcomes using positive verbal instructions (e.g., 'I expect your pain will improve after this manipulation').	☐	☐	☐	☐

*4.***What is your opinion of attempting to reduce *negative outcomes* via the following approaches?**

	**Please tick applicable box(es)** *Required*			
	a) I think it is a valid approach	b) I use this approach in practice	c) I am confident to use without training	Not Applicable
1. Reinforcing a shift in patient's negative thoughts to positive ones (e.g., outcomes to highlight progress).	☐	☐	☐	☐
2. Rephrasing negative information (e.g., during leg flexion test: 'this procedure may lead to a slight increase in pain' rather say instead: 'this procedure might be a bit uncomfortable but only temporarily').	☐	☐	☐	☐
3. Describing how (un)common side effects are numerically (e.g., 1 in 100 people).	☐	☐	☐	☐
4. Anticipating and helping reduce patient's anxiety about the treatment / procedure.	☐	☐	☐	☐
5. Allocating time for patients to ask about negative aspects of treatment.	☐	☐	☐	☐
6. Avoiding negative phrases (e.g., 'wear and tear', 'damage', 'degeneration', 'ongoing' instead of 'chronic' pain, 'plan activities' instead of 'do exercise').	☐	☐	☐	☐

Page 5: (1) Patient's Beliefs and Characteristics continued

Please ***select / tick all applicable column(s)*** if:
a) You believe the statement below reflects a potentially valid care approach;
b) It is an approach/technique you currently use as part of your everyday practice;
c) It is an approach/technique you feel confident to use without further training / experience.

---

5. What is your opinion of the following *cognitive behavioural strategies*?

	**Please tick applicable box(es)** *Required*			
	a) I think it is a valid approach	b) I use this approach in practice	c) I am confident to use without training	Not Applicable
1. Reframing patient's prior misconceptions about low back pain (e.g., 'pain is not always a sign of physical tissue damage', 'your spine is flexible not fragile').	☐	☐	☐	☐
2. Reframing patient's prior misconceptions about treatment (e.g., 'bed rest does not usually help patients recover faster but modified activity can').	☐	☐	☐	☐
3. Explaining the multi-dimensional nature (biopsychosocial aspects) of pain (i.e., beliefs, emotions, and behaviours (movement and lifestyle)) via suitable educational materials.	☐	☐	☐	☐
4. Clarifying maladaptive perceptions (e.g., catastrophising: 'My vertebrae are out of line. I stopped gardening, so I won't end up in wheelchair').	☐	☐	☐	☐
5. Assisting in decreasing fear-avoidance and harm beliefs along with avoidant behaviours.	☐	☐	☐	☐
6. Helping patients plan and monitor treatment success (e.g., SMART goals, motivational interviewing).	☐	☐	☐	☐
7. Empowering patients to self-care and anticipate barriers (e.g., reminders, implementation intentions, journal / logbook, NHS online self-care resources).	☐	☐	☐	☐
8. Developing patient's self-confidence in performing and persisting with a new behaviour to pursue a goal.	☐	☐	☐	☐

6. **What is your opinion of considering *sociocultural contexts*?**

	**Please tick applicable box(es)** *Required*			
	a) I think it is a valid approach	b) I use this approach in practice	c) I am confident to use without training	Not Applicable
1. Displaying a balanced attitude to patient's alternative or cultural beliefs if not harmful (e.g., acupuncture).	☐	☐	☐	☐
2. Involving significant others and/or primary carers in treatment.	☐	☐	☐	☐

Optional:

Please provide any additional ideas / suggestions concerning **patient's characteristics and beliefs** you may have based on your expertise, and practice with chronic LBP patients **and select the relevant checkbox(es)**.

*7.*Please specify any further suggestions:

		**Please tick applicable box(es)**		
	Optional	a) I think it is a valid approach	b) I use this approach in practice	c) I am confident to use without training
a)				
b)				
c)				
d)				
e)				

Page 6: (2) Practitioner's Beliefs and Characteristics

Please ***select / tick all applicable column(s)*** if:
a) You believe the statement below reflects a potentially valid care strategy;
b) It is a care strategy you currently use as part of your everyday practice;
c) You believe this care strategy might contribute to or enhance overall treatment effects.

---

*8.*What is your opinion of demonstrating *your expertise* via the following approaches?

	**Please tick applicable box(es)** *Required*			
	a) I think it is a valid care strategy	b) I use this care strategy in practice	c) It might enhance treatment effects	Not Applicable
1. Prescribing or administering treatments you believe and expect to be effective.	☐	☐	☐	☐
2. Clearly communicating your expectations (i.e., what you anticipate will occur) whilst administering care.	☐	☐	☐	☐
3. Using indicators of expertise / high status (e.g., health qualifications, professional memberships) in offices or correspondence.	☐	☐	☐	☐
4. Wearing a laboratory coat / medical apparel or tailored / formal clothing to symbolise professionalism.	☐	☐	☐	☐

*9.*What is your opinion of adapting your *mindset* or *attitude* via the following approaches?

	**Please tick applicable box(es)** *Required*			
	a) I think it is a valid care strategy	b) I use this care strategy in practice	c) It might enhance treatment effects	Not Applicable
1. Remaining attentive and fully focused on the patient throughout the appointment.	☐	☐	☐	☐
2. Being genuine and honest to instil a sense of trustworthiness and authenticity.	☐	☐	☐	☐
3. Displaying self-confidence without appearing arrogant or dismissive.	☐	☐	☐	☐

Optional:

Please provide any additional ideas / suggestions concerning **practitioner's characteristics and beliefs** you may have based on your expertise, and practice with chronic LBP patients **and select the relevant checkbox(es)**.

*10.***Please specify any further suggestions:**

		**Please tick applicable box(es)**		
	Optional	a) I think it is a valid approach	b) I use this approach in practice	c) It might enhance treatment effects
a)				
b)				
c)				
d)				
e)				

<u>Page 7: (3) Patient–Practitioner Relationship</u>

Please ***select / tick all applicable column(s)*** if:
a) You believe the statement below reflects a potentially valid care approach;
b) It is an approach/technique you currently use as part of your everyday practice;
c) It is an approach/technique you feel confident to use without further training / experience.

---

*11.*What is your opinion of displaying the following *non-verbal behaviours*?

	**Please tick applicable box(es)** *Required*			
	a) I think it is a valid approach	b) I use this approach in practice	c) I am confident to use without training	Not Applicable
1. Being warm, confident, friendly, relaxed and open during the appointment.	☐	☐	☐	☐
2. Using eye contact, smiling, caring expressions of support and interest to convey empathy and compassion.	☐	☐	☐	☐
3. Using affirmative head nodding, forward leaning, open body postures / orientations.	☐	☐	☐	☐
4. Not rushing or interrupting the patient; giving them time to tell their story.	☐	☐	☐	☐
5. Applying different forms of touch (e.g., assistive touch, touch to prepare the patient, touch to provide information, touch to reassure the patient).	☐	☐	☐	☐

*12.*What is your opinion of the following aspects of the *patient-centred approach*?

	**Please tick applicable box(es)** *Required*			
	a) I think it is a valid approach	b) I use this approach in practice	c) I am confident to use without training	Not Applicable
1. Using verbal expressions of empathy, support, and language reciprocity (e.g., using the patient's words / phrasing).	☐	☐	☐	☐
2. Ensuring the patient feels listened to and heard (e.g., active listening or noting their responses).	☐	☐	☐	☐
3. Adopting psychosocial talk or partnership statements (e.g., we, us, together).	☐	☐	☐	☐

	a) I think it is a valid approach	b) I use this approach in practice	c) I am confident to use without training	Not Applicable
4. Requesting the patient’s opinions and demonstrating you trust and respect them.	☐	☐	☐	☐
5. Individualising the interaction style according to a patient’s preference (e.g., collaborative or authoritative).	☐	☐	☐	☐
6. Providing treatment choices and encouraging patients to choose option(s) if they so wish.	☐	☐	☐	☐
7. Engaging in collaborative decision-making with patients (e.g., mutually agreed and flexible goals).	☐	☐	☐	☐
8. Promoting the patient’s sense of relatedness and partnership with you (i.e., therapeutic alliance).	☐	☐	☐	☐

13. **What is your opinion of using the following *diagnostic practices*?**

	**Please tick applicable box(es)** *Required*			
	a) I think it is a valid approach	b) I use this approach in practice	c) I am confident to use without training	Not Applicable
1. Providing a detailed, definitive, and confident diagnosis.	☐	☐	☐	☐
2. Providing effective reassurance via clear and understandable explanations.	☐	☐	☐	☐
3. Asking questions about the meaning of the patient’s symptoms (i.e., what symptoms indicate to them).	☐	☐	☐	☐
4. Examining the patient fully using appropriate therapeutic ‘hands on’ touch during the clinical examination.	☐	☐	☐	☐

Optional:

Please provide any additional ideas / suggestions concerning **the patient-practitioner relationship** you may have based on your expertise, and practice with chronic LBP patients **and select the relevant checkbox(es)**.

*14.***Please specify any further suggestions:**

		**Please tick applicable box(es)**		
	Optional	a) I think it is a valid approach	b) I use this approach in practice	c) I am confident to use without training
a)				
b)				
c)				
d)				
e)				

Page 8: (4) Treatment Characteristics

Please ***select / tick all applicable column(s)*** if:
a) You believe the statement below reflects a potentially valid care strategy;
b) It is a care strategy you currently use as part of your everyday practice;
c) You believe this care strategy might contribute to or enhance overall treatment effects.

---

15. What is your opinion when explaining the following *treatment options*?

	**Please tick applicable box(es)** *Required*			
	a) I think it is a valid care strategy	b) I use this care strategy in practice	c) It might enhance treatment effects	Not Applicable
1. Overtly encouraging patients to engage in therapy / exercise with an optimistic mindset to try establish positive associations with pain relief.	☐	☐	☐	☐
2. Encouraging patients to find suitable incentives / reinforcement strategies to increase daily activity (e.g., personalised activities, exercise partners).	☐	☐	☐	☐
3. To show and tell the patient that as a therapy is applied it helps (e.g. 'I am applying pressure here because it helps…').	☐	☐	☐	☐

16. What is your opinion of the following *appointment features*?

	**Please tick applicable box(es)** *Required*			
	a) I think it is a valid care strategy	b) I use this care strategy in practice	c) It might enhance treatment effects	Not Applicable
1. Ensuring the patient is cared for by the same practitioner / therapist (i.e., continuity of care).	☐	☐	☐	☐
2. Verbalising future treatment plans by stating the number of appointments and/or follow-ups (e.g., 'I will treat you every second week for 30 minutes').	☐	☐	☐	☐
3. Increasing the frequency and/or duration of appointments (i.e., provide extra time / attention).	☐	☐	☐	☐

17. **What is your opinion of the following *alternative feedback* strategies?**

	**Please tick applicable box(es)** *Required*			
	a) I think it is a valid care strategy	b) I use this care strategy in practice	c) It might enhance treatment effects	Not Applicable
1. Administering treatments along with visual feedback (e.g., using mirrors during exercises).	☐	☐	☐	☐
2. Enabling patients to engage with other patients undergoing treatment with positive results (e.g., group exercise classes, sharing success stories / testimonials, informally in the waiting area).	☐	☐	☐	☐

Optional:
Please provide any additional ideas / suggestions concerning **the treatment characteristics** you may have based on your expertise, and practice with chronic LBP patients **and select the relevant checkbox(es)**.

18. **Please specify any further suggestions:**

		**Please tick applicable box(es)**		
	Optional	a) I think it is a valid approach	b) I use this approach in practice	c) It might enhance treatment effects
a)				
b)				
c)				
d)				
e)				

Page 9: (5) Healthcare Setting / Environment

Please ***select / tick all applicable column(s)*** if:
a) You believe the statement below reflects a potentially valid care strategy;
b) It is a care strategy you currently use as part of your everyday practice;
c) You believe this care strategy might contribute to or enhance overall treatment effects.

---

*19.*What is your opinion of the following *interior design and layout* strategies?

	**Please tick applicable box(es)** *Required*			
	a) I think it is a valid care strategy	b) I use this care strategy in practice	c) It might enhance treatment effects	Not Applicable
1. Ensuring facilities have ample natural light or windows, and are suitably heated / ventilated (i.e., comfortable temperature).	☐	☐	☐	☐
2. Ensuring treatment facilities have privacy provisions (e.g., private changing area and treatment room, curtains / blinds on windows).	☐	☐	☐	☐
3. Considering seating provisions in the waiting areas (e.g., quantity, varying chair sizes, general arrangement).	☐	☐	☐	☐
4. Considering seating provisions in treatment office (e.g., relative position to desk, additional chairs for carer).	☐	☐	☐	☐

*20.*What is your opinion of the *setting's decor*?

	**Please tick applicable box(es)** *Required*			
	a) I think it is a valid care strategy	b) I use this care strategy in practice	c) It might enhance treatment effects	Not Applicable
1. Waiting areas and treatment facilities are uncluttered and tidy.	☐	☐	☐	☐
2. Providing visual indicators or cues to signify it is a medical setting (e.g., model of spine, patient information brochures, medicalised décor).	☐	☐	☐	☐
3. Decorating the waiting area with cheerful ornamentation (e.g., healthy indoor plants, leisure reading materials, comfortable cushions).	☐	☐	☐	☐

	a) I think it is a valid care strategy	b) I use this care strategy in practice	c) It might enhance treatment effects	Not Applicable
4. Combining positive distractors such as soft or soothing music, nice aromas, hot or cold beverages.	☐	☐	☐	☐
5. Using nature artworks that include green vegetation, flowers, or water may help to reduce anxiety.	☐	☐	☐	☐

Optional:
Please provide any additional ideas / suggestions concerning **the healthcare environment** you may have based on your expertise, and practice with chronic LBP patients **and select the relevant checkbox(es)**.

*21.***Please specify any further suggestions:**

		**Please tick applicable box(es)**		
	Optional	a) I think it is a valid approach	b) I use this approach in practice	c) It might enhance treatment effects
a)				
b)				
c)				
d)				
e)				

*22.***Age:** (years)

Please enter a number.

[ ]

*23.***Gender:** *(please select option from drop-down menu)*

[ ]

(drop-down menu)
Male
Female
Non-binary gender
Prefer not to disclose
Other

a. If you selected Other, please specify:

[ ]

*24.***Practitioner Type:** *(please select one option) Required*

- ○ Chiropractor
- ○ Osteopath
- ○ Physiotherapist
- ○ Sports Therapist
- ○ Prefer not to disclose
- ○ Other

a. If you selected Other, please specify:

[ ]

*25.***Years of Clinical Experience** (Post-Qualifying)**:** (years)

Please enter a number.

[ ]

*26.***Current Practice Setting:** *(please select one option)*

- ○ Private practice
- ○ Public (NHS setting)
- ○ Combination of both
- ○ Prefer not to disclose
- ○ Other

a. If you selected Other, please specify:

[ ]

*27.***Current Practice Region:** *(please select option from drop-down menu) Required*

(drop-down menu)
Northern Ireland
Scotland
Wales
North East and Cumbria
North West
Yorkshire and the Humber
West Midlands
East Midlands
London
East of England
South East
South West
Prefer not to disclose
Other

a. If you selected Other, please specify:

Page 11: Expression of Interest

Second Panel-Round

If you might be **interested in participating in the second round** of this Delphi-study, please **include your email address** in the textbox below:

*28.**Please DO NOT provide your NHS email address:** Optional*

Please enter a valid email address.

Note:

- An invitation will be sent to you within the next six to eight months (i.e., between June and August 2020), providing information on the second panel-round, and inviting you to take part.
- In the interim, your email address will be securely stored on this password protected online survey platform (**hosted by Jisc**: https://www.onlinesurveys.ac.uk/).
- Jisc acts as the Data Processor, and is both GDPR compliant and ISO 27001 certified.
- Once the second set of invitations / follow-ups emails are sent out, your email address will be confidentially destroyed.
- Only Bronwyn Sherriff (primary researcher) has direct access to the raw (non-anonymised) survey responses collected via Jisc.
- **All raw data will be anonymised, and any personal information identifying factors removed, prior to such information being exported from the Jisc platform or being shared.**

Page 12: Closing Message

- **Thank you** for choosing to take part in this survey.
- I am truly **grateful for your time** and invaluable insights.
- If you have **colleagues** who may also be **interested in participating**, **please** may I ask you to **forward the survey link**.

Researcher's Contact Details:
Please do not hesitate to contact me if you have any queries or would like to be kept updated on the findings of this study.

Ms Bronwyn Sherriff: bsherriff@bournemouth.ac.uk
Alternatively, you are welcome to contact one of my PhD supervisors instead:

- Prof. Carol Clark: cclark@bournemouth.ac.uk
- Prof. David Newell: dnewell@aecc.ac.uk
- Dr Clare Killingback: c.killingback@hull.ac.uk

Copy of Delphi Survey – Round 2 (DS-R2)

Page 1: Welcome and Introduction

Welcome to the second round of this Delphi-consensus survey.

- Thank you for your invaluable input on the previous survey, it has informed the development of the second round of this Delphi-study.
- This research seeks to investigate manual and physical therapists' perceptions of the influence of five main types of contextual factors during the management of patients with chronic or persistent low back pain (LBP), namely:

1. **practitioner's beliefs and characteristics** (e.g., beliefs, expertise, appearance);
2. **patient's beliefs and characteristics** (e.g., beliefs, expectations, previous experiences);
3. **patient-practitioner relationship** (e.g., overt communication, patient-centred approach);
4. **treatment features / characteristics** (e.g., overt therapy, appointment features);
5. **treatment environment / setting** (e.g., layout, interior design).

Next Steps:

- If you are willing to participate, please select the **Consent Statement checkboxes** at the bottom of the page **and then *'next'*** to begin.
- The questionnaire takes approximately **15 to 20 minutes** to complete.
- You may select the ***'finish later'*** option and email yourself a link or leave the browser window open to continue at a later time.
- Survey **responses are collected over encrypted SSL** (TLS) connections to ensure information is transmitted securely.
- You will be able to provide your email address at the end of the questionnaire if you would like to receive a summary of the findings.

Researcher's Contact Details:
This Delphi-study forms part of a broader research project which is being conducted in order to obtain a PhD qualification from Bournemouth University, in partnership with AECC University College.

Please do not hesitate to contact me if you have any additional questions or specific concerns.
Ms Bronwyn Sherriff: bsherriff@bournemouth.ac.uk
Alternatively, you are welcome to contact any one of my PhD supervisors:

- Prof. Carol Clark: cclark@bournemouth.ac.uk
- Prof. David Newell: dnewell@aecc.ac.uk
- Dr Clare Killingback: c.killingback@hull.ac.uk

*1.***Consent to Participate** *Required*

☐ I confirm that I have read and understood the information provided.

☐ I agree to take part in the study on the basis set out in the Information Sheet provided to me via email.

Page 2: Demographics

*2.***Age:** (years) *Required*

Please enter a number.

[ ]

*3.***Gender:** *(please select option from drop-down menu) Required*

[ ]

(drop-down menu)

Male

Female

Non-binary

Prefer not to disclose

Other

a. If you selected Other, please specify:

[ ]

*4.***Practitioner Type:** *(please select one option) Required*

- ○ Chiropractor
- ○ Osteopath
- ○ Physiotherapist
- ○ Sports Therapist
- ○ Other

a. If you selected Other, please specify:

[ ]

*5.***Years of Clinical Experience** (Post-Qualifying)**:** (years) *Required*

Please enter a number.

[ ]

*6.***Practice Setting:** *(please select one option)*

Note: The following question relates to your **pre-COVID-19 practice setting** rather than how you may currently be practising.

- ○ Private practice
- ○ Public (NHS setting)
- ○ Combination of both
- ○ Educational organisation
- ○ Charity / Non-profit organisation
- ○ Other

a. If you selected Other, please specify:

[ ]

*7.***Current Practice Region:** *(please select option from drop-down menu) Required*

(drop-down menu)

Northern Ireland
Scotland
Wales
North East and Cumbria
North West
Yorkshire and the Humber
West Midlands
East Midlands
London
East of England
South East
South West
Other

a. If you selected Other, please specify:

Page 3: (1) Practitioner's Beliefs and Characteristics

- Below is a list of care approaches for patients with chronic or persistent low back pain (LBP).
- Please indicate whether you **have intentionally used** each approach **believing it could influence patient's** LBP **outcome(s)**.

- Select **1** or **2** if you **did not believe** it could improve outcome(s).
- Select **3** if you were **unsure** if it could improve outcome(s).
- Select **4** or **5** if you **believed it could** improve outcome(s).
- Select '**Not Valid**' if you **do not think it is a suitable approach** for patients with chronic LBP.

*8.***Please indicate how much you agree or disagree with the influence of each approach on patient's outcome(s).** *Required*

	**1 – Strongly Disagree**	**2 – Disagree**	**3 – Neither Agree nor Disagree**	**4 – Agree**	**5 – Strongly Agree**	**Not Valid**	**Do Not Recall / Use**
1. Remaining attentive and fully focused on the patient throughout the appointment.							
2. Being genuine and honest to instil a sense of trustworthiness and authenticity.							
3. Displaying self-confidence without appearing dismissive.							
4. Being calm and compassionate throughout the appointment.							
5. Displaying a professional and caring (not only "curing") attitude.							
6. Creating a caring atmosphere (e.g., appear to have all the time in the world; ensure each patient feels like a priority).							
7. Actively build rapport with each patient (e.g., discuss common interests / hobbies; enquire about their lives).							
8. Administering treatments you expect to be effective.							
9. Clearly communicating your expectations (i.e., what you anticipate will occur) whilst administering care.							
10. Using indicators to display your expertise or credibility (e.g., qualifications, insurance, professional memberships) in reception / office, website, or correspondence.							
11. Demonstrating professionalism through your general appearance (i.e., being clean, tidy, smart, and presentable).							

Page 4: (2) Patient's Beliefs and Characteristics

- Below is a list of care approaches for patients with chronic or persistent low back pain (LBP).
- Please indicate whether you **have intentionally used** each approach **believing it could influence patient's** LBP **outcome(s)**.

- Select **1** or **2** if you **did not believe** it could improve outcome(s).
- Select **3** if you were **unsure** if it could improve outcome(s).
- Select **4** or **5** if you **believed it could** improve outcome(s).
- Select '**Not Valid**' if you **do not think it is a suitable approach** for patients with chronic LBP.

9.**Please indicate how much you agree or disagree with the influence of each approach on patient's outcome(s).** *Required*

	**1 – Strongly Disagree**	**2 – Disagree**	**3 – Neither Agree nor Disagree**	**4 – Agree**	**5 – Strongly Agree**	**Not Valid**	**Do Not Recall / Use**
1. Actively investigating patient's needs, feelings, preferences, and previous experiences.	☐	☐	☐	☐	☐	☐	☐
2. Supporting the patient in reframing negative memories (e.g., reinterpret an x-ray / scan or explain radiology reports / GP letters).	☐	☐	☐	☐	☐	☐	☐
3. Reframing misinformed beliefs from previous healthcare experiences (e.g., 'my spine is crumbling', 'my spinal curve is abnormal', 'my back is worn out').	☐	☐	☐	☐	☐	☐	☐
4. Exploring the patient's current or pre-existing beliefs about the cause(s) of their LBP.	☐	☐	☐	☐	☐	☐	☐
5. Communicating an intervention is likely to be effective using positive verbal instructions (e.g., 'I expect your pain will improve after treatment').	☐	☐	☐	☐	☐	☐	☐
6. Emphasising positive outcomes such as overall pain-reducing effects (e.g., 'manual or physical therapies are often as effective as painkillers').	☐	☐	☐	☐	☐	☐	☐
7. Being optimistic during treatment by providing a prognosis (e.g., 'I believe you will recover and get back to your usual level of functioning').	☐	☐	☐	☐	☐	☐	☐
8. Instilling genuine hope in patients regarding how their life can change for the better.	☐	☐	☐	☐	☐	☐	☐

- Select **1** or **2** if you **did not believe** it could improve outcome(s).
- Select **3** if you were **unsure** if it could improve outcome(s).
- Select **4** or **5** if you **believed it could** improve outcome(s).
- Select '**Not Valid**' if you **do not think it is a suitable approach** for patients with chronic LBP.

*10.***Please indicate how much you agree or disagree with the influence of each approach on patient's outcome(s).** *Required*

	**1 – Strongly Disagree**	**2 – Disagree**	**3 – Neither Agree nor Disagree**	**4 – Agree**	**5 – Strongly Agree**	**Not Valid**	**Do Not Recall / Use**
1. Reinforcing a shift in patient's negative thoughts to positive ones (e.g., monitor outcomes to highlight progress).	☐	☐	☐	☐	☐	☐	☐
2. Rephrasing negative information (e.g., leg flexion test: 'this procedure might be a bit uncomfortable but only temporarily').	☐	☐	☐	☐	☐	☐	☐
3. Using simple, everyday analogies to alter patient's negative illness perceptions (e.g., rusty hinges often work well despite their appearance).	☐	☐	☐	☐	☐	☐	☐
4. Anticipating and helping reduce patient's anxiety about the treatment / procedure.	☐	☐	☐	☐	☐	☐	☐
5. Allocating time for patients to ask about negative aspects of treatment to address their concerns openly and honestly.	☐	☐	☐	☐	☐	☐	☐
6. Avoiding negative phrases (e.g., 'wear and tear', 'damage', 'degeneration', 'abnormal').	☐	☐	☐	☐	☐	☐	☐
7. Explaining that calming their stress response is a part of everyday self-care for physical pain and healing.	☐	☐	☐	☐	☐	☐	☐
8. Explaining imaging is usually unnecessary because scans may not explain the extent of their pain and/or dysfunction.	☐	☐	☐	☐	☐	☐	☐

- Select **1** or **2** if you **did not believe** it could improve outcome(s).
- Select **3** if you were **unsure** if it could improve outcome(s).
- Select **4** or **5** if you **believed it could** improve outcome(s).
- Select '**Not Valid**' if you **do not think it is a suitable approach** for patients with chronic LBP.

*11.***Please indicate how much you agree or disagree with the influence of each approach on patient's outcome(s).**

	**1 – Strongly Disagree**	**2 – Disagree**	**3 – Neither Agree nor Disagree**	**4 – Agree**	**5 – Strongly Agree**	**Not Valid**	**Do Not Recall / Use**
1. Reframing patient's prior misconceptions about their anatomy / physiology (e.g., 'your spine is flexible not fragile').	☐	☐	☐	☐	☐	☐	☐
2. Reframing patient's prior misconceptions about treatment (e.g., 'bed rest does not usually help patients recover faster but modified activity can').	☐	☐	☐	☐	☐	☐	☐
3. Explaining the multi-dimensional nature (biopsychosocial aspects) of pain (i.e., beliefs, emotions, and behaviours (movement and lifestyle)) via suitable educational materials.	☐	☐	☐	☐	☐	☐	☐
4. Explaining basic pain science (i.e., perceived pain is not necessarily actual physical pain from nerve or tissue damage, but whilst very real, is more of a 'learned' response to prior experiences).	☐	☐	☐	☐	☐	☐	☐
5. Explaining routine activities, movement, or exercise can help 'rewire' perceived pain pathways (e.g., some pain or discomfort is normal but is not a sign their LBP is "worsening").	☐	☐	☐	☐	☐	☐	☐
6. Clarifying maladaptive perceptions (e.g., catastrophising: 'My vertebrae are out of line. I stopped gardening, so I won't end up in a wheelchair').	☐	☐	☐	☐	☐	☐	☐
7. Assisting in decreasing fear-avoidance and harm beliefs by recognising, confronting, and correcting them.	☐	☐	☐	☐	☐	☐	☐
8. Helping patients plan and monitor treatment success (e.g., explain outcome measures; co-create short-term and long-term goals or target-driven stages of improvement).	☐	☐	☐	☐	☐	☐	☐
9. Developing patient's self-confidence in performing or persisting with a new behaviour or goal.	☐	☐	☐	☐	☐	☐	☐

Page 5: (3) Patient–Practitioner Relationship

- Below is a list of care approaches for patients with chronic or persistent low back pain (LBP).
- Please indicate whether you **have intentionally used** each approach **believing it could influence patient's** LBP **outcome(s)**.

- Select **1** or **2** if you **did not believe** it could improve outcome(s).
- Select **3** if you were **unsure** if it could improve outcome(s).
- Select **4** or **5** if you **believed it could** improve outcome(s).
- Select '**Not Valid**' if you **do not think it is a suitable approach** for patients with chronic LBP.

12. **Please indicate how much you agree or disagree with the influence of each approach on patient's outcome(s).** *Required*

	1 – Strongly Disagree	2 – Disagree	3 – Neither Agree nor Disagree	4 – Agree	5 – Strongly Agree	Not Valid	Do Not Recall / Use
1. Being warm, friendly, and relaxed during the appointment.	☐	☐	☐	☐	☐	☐	☐
2. Using eye contact, smiling, caring expressions of support to convey empathy or compassion.	☐	☐	☐	☐	☐	☐	☐
3. Using affirmative head nodding, forward leaning, open body postures / orientations.	☐	☐	☐	☐	☐	☐	☐
4. Not rushing or interrupting the patient; giving them time to tell their story.	☐	☐	☐	☐	☐	☐	☐
5. Applying different forms of touch (e.g., assistive touch, touch to prepare the patient, touch to provide information, touch to reassure the patient).	☐	☐	☐	☐	☐	☐	☐
6. Providing a confident diagnosis (e.g., providing a diagram with simple explanations and/or notes).	☐	☐	☐	☐	☐	☐	☐
7. Explaining improvement(s) can be dynamic, and their condition / symptoms may change throughout treatment.	☐	☐	☐	☐	☐	☐	☐
8. Providing a meaningful explanation of the patient's LBP (i.e., cognitive reassurance) which is clear, understandable, and can be referred to after treatment.	☐	☐	☐	☐	☐	☐	☐
9. Asking questions about the meaning of the patient's symptoms (i.e., what symptoms indicate to them).	☐	☐	☐	☐	☐	☐	☐
10. Examining the patient fully using appropriate therapeutic 'hands on' touch during the clinical examination.	☐	☐	☐	☐	☐	☐	☐

- Select **1** or **2** if you **did not believe** it could improve outcome(s).
- Select **3** if you were **unsure** if it could improve outcome(s).
- Select **4** or **5** if you **believed it could** improve outcome(s).
- Select '**Not Valid**' if you **do not think it is a suitable approach** for patients with chronic LBP.

*13.***Please indicate how much you agree or disagree with the influence of each approach on patient's outcome(s).** *Required*

	**1 – Strongly Disagree**	**2 – Disagree**	**3 – Neither Agree nor Disagree**	**4 – Agree**	**5 – Strongly Agree**	**Not Valid**	**Do Not Recall / Use**
1. Using verbal expressions of empathy, support, and language reciprocity (e.g., using the patient's words).	☐	☐	☐	☐	☐	☐	☐
2. Compassionately expressing your understanding of how LBP affects them (e.g., 'I understand how frustrating it is not to be able to walk your dog / go dancing / garden' etc).	☐	☐	☐	☐	☐	☐	☐
3. Ensuring the patient feels listened to and heard (e.g., active listening or noting their responses).	☐	☐	☐	☐	☐	☐	☐
4. Adopting psychosocial talk or partnership statements (e.g., we, us, together).	☐	☐	☐	☐	☐	☐	☐
5. Demonstrating you trust or respect the patient and their opinions.	☐	☐	☐	☐	☐	☐	☐
6. Individualising the interaction style according to a patient's preference (e.g., collaborative or authoritative).	☐	☐	☐	☐	☐	☐	☐
7. Engaging in collaborative decision-making together (e.g., mutually agreed and flexible goals).	☐	☐	☐	☐	☐	☐	☐
8. Promoting the patient's sense of relatedness and partnership with you (i.e., therapeutic alliance).	☐	☐	☐	☐	☐	☐	☐
9. Confirming the patient not only heard but also understood the content of your communication.	☐	☐	☐	☐	☐	☐	☐

Page 6: (4) Treatment Characteristics

- Below is a list of care approaches for patients with chronic or persistent low back pain (LBP).
- Please indicate whether you **have intentionally used** each approach **believing it could influence patient's** LBP **outcome(s)**.

- Select **1** or **2** if you **did not believe** it could improve outcome(s).
- Select **3** if you were **unsure** if it could improve outcome(s).
- Select **4** or **5** if you **believed it could** improve outcome(s).
- Select '**Not Valid**' if you **do not think it is a suitable approach** for patients with chronic LBP.

*14.***Please indicate how much you agree or disagree with the influence of each approach on patient's outcome(s).** *Required*

	**1 – Strongly Disagree**	**2 – Disagree**	**3 – Neither Agree nor Disagree**	**4 – Agree**	**5 – Strongly Agree**	**Not Valid**	**Do Not Recall / Use**
1. Overtly encouraging patients to engage in therapy / exercise with an optimistic mindset to try establish positive associations with pain relief.	☐	☐	☐	☐	☐	☐	☐
2. Encouraging patients to find suitable incentives / reinforcement strategies to increase daily activity (e.g., personalised activities, exercise partners).	☐	☐	☐	☐	☐	☐	☐
3. Clearly explaining the difference between a clinical examination and treatment.	☐	☐	☐	☐	☐	☐	☐
4. Demonstrating whether functional change has occurred immediately after treatment (e.g., pain, range of motion, or strength).	☐	☐	☐	☐	☐	☐	☐
5. Explaining your treatment advice in line with the patient's treatment expectations.	☐	☐	☐	☐	☐	☐	☐
6. Ensuring the patient is cared for by the same practitioner / therapist (i.e., continuity of care).	☐	☐	☐	☐	☐	☐	☐
7. Increasing the frequency and/or duration of appointments (i.e., provide extra time / attention).	☐	☐	☐	☐	☐	☐	☐
8. Providing patients with clear milestones or signposting to indicate their progression through the treatment programme.	☐	☐	☐	☐	☐	☐	☐

	1 – Strongly Disagree	2 – Disagree	3 – Neither Agree nor Disagree	4 – Agree	5 – Strongly Agree	Not Valid	Do Not Recall / Use
9. Administering treatments along with visual feedback (e.g., using mirrors during exercises).	☐	☐	☐	☐	☐	☐	☐
10. Providing self-management materials (e.g., videos, rehabilitation booklets) or email / telephone support to promote a patient's engagement in physical activities.	☐	☐	☐	☐	☐	☐	☐
11. Displaying feedback from other patients to provide reassurance (i.e., testimonials displayed on TV in waiting area, or online via website).	☐	☐	☐	☐	☐	☐	☐
12. Sharing positive stories of other (anonymous) patients with similar problems or goals.	☐	☐	☐	☐	☐	☐	☐

Page 7: (5) Treatment Environment / Setting

- Below is a list of care approaches for patients with chronic or persistent low back pain (LBP).
- Please indicate whether you **have intentionally used** each approach **believing it could influence patient's** LBP **outcome(s)**.

- Select **1** or **2** if you **did not believe** it could improve outcome(s).
- Select **3** if you were **unsure** if it could improve outcome(s).
- Select **4** or **5** if you **believed it could** improve outcome(s).
- Select '**Not Valid**' if you **do not think it is a suitable approach** for patients with chronic LBP.

*15.***Please indicate how much you agree or disagree with the influence of each approach on patient's outcome(s).** *Required*

	**1 – Strongly Disagree**	**2 – Disagree**	**3 – Neither Agree nor Disagree**	**4 – Agree**	**5 – Strongly Agree**	**Not Valid**	**Do Not Recall / Use**
1. Ensuring treatment facilities have ample natural light or windows, and are suitably heated / ventilated (i.e., comfortable temperature).	☐	☐	☐	☐	☐	☐	☐
2. Ensuring treatment facilities have privacy provisions (e.g., private changing area and treatment room, curtains / blinds on windows).	☐	☐	☐	☐	☐	☐	☐
3. Rearranging the furniture or seating provisions in the treatment office (e.g., relative position to desk, additional chairs for carer).	☐	☐	☐	☐	☐	☐	☐
4. Ensuring waiting areas and treatment facilities are uncluttered and tidy.	☐	☐	☐	☐	☐	☐	☐
5. Providing visual indicators or cues to signify it is a medical setting (e.g., model of spine, patient information brochures, medicalised décor).	☐	☐	☐	☐	☐	☐	☐
6. Creating a positive ambience or atmosphere (e.g., flowers, plants, interesting magazines, friendly staff, relaxing background music, warm lighting).	☐	☐	☐	☐	☐	☐	☐
7. Using nature artworks that include green vegetation, flowers, or water features.	☐	☐	☐	☐	☐	☐	☐

*16.*On a scale ranging from **1 (no control)** to **6 (full control)**, please indicate how much personal control or input you have on the overall layout and design of the treatment room (i.e., usual care setting **prior to the COVID-19 pandemic**). *Required*

- 1 – No control
- 2 – Almost no control
- 3 – Little control
- 4 – Some control
- 5 – Almost full control
- 6 – Full control
- Not Applicable

Page 8: Contextual Factors

*17.*On a scale ranging from **1 (not at all important)** to **7 (extremely important)**, based on your experience and beliefs, please **rate the importance of each contextual factor to the patient's treatment** during the healthcare encounter. *Required*

Higher ratings indicate you believe the contextual factor is important.
You may choose to select the same rating for different contextual factors.

	**1 – Not at all important**	**2 – Low importance**	**3 – Slightly important**	**4 – Neutral**	**5 – Moderately important**	**6 – Very important**	**7 – Extremely important**
Practitioner’s beliefs and characteristics (e.g., beliefs, expertise, appearance)	☐	☐	☐	☐	☐	☐	☐
Patient’s beliefs and characteristics (e.g., beliefs, expectations, previous experiences)	☐	☐	☐	☐	☐	☐	☐
Patient-practitioner relationship (e.g., overt communication, patient-centred approach)	☐	☐	☐	☐	☐	☐	☐
Treatment features / characteristics (e.g., overt therapy, appointment features)	☐	☐	☐	☐	☐	☐	☐
Treatment environment / setting (e.g., layout, interior design)	☐	☐	☐	☐	☐	☐	☐

a. Please **explain why** you have chosen the above ratings. *Optional*

*18.*Based on your experience and beliefs, please indicate which contextual factor you feel is the **most important to the patient's treatment** during the healthcare encounter. *Required*

(drop down menu)
Practitioner's beliefs and characteristics
Patient's beliefs and characteristics
Patient-practitioner relationship
Treatment features / characteristics
Treatment environment / setting

*19.*Based on your experience and beliefs, please indicate which contextual factor you feel is the **least important to the patient's treatment** during the healthcare encounter. *Required*

(drop down menu)
Practitioner's beliefs and characteristics
Patient's beliefs and characteristics
Patient-practitioner relationship
Treatment features / characteristics
Treatment environment / setting

Page 9: Consultation Approach

Note: The following questions relate to your **pre-COVID-19 consultation approach** rather than how you may currently be practising during the global pandemic.

Although there are a range of consultation approaches and styles, which may differ depending on the patient and context, please try to place yourself on the continuum below.

*20.*On a scale ranging from **mainly hands-on** (i.e., biomechanical orientation) to **mainly hands-off** (i.e., psychosocial orientation), please **rate your typical engagement style** during the treatment of patients with chronic or persistent LBP. *Required*

- ○ 10 – Mainly hands-off
- ○ 9
- ○ 8
- ○ 7
- ○ 6
- ○ 5 – Combined approach
- ○ 4
- ○ 3
- ○ 2
- ○ 1
- ○ 0 – Mainly hands-on

a. Please **explain why** you have chosen the above rating. *Optional*

*21.*Please select any of the following factors which you believe has **mainly influenced** or shaped **your consultation approach**. *Required*

Please select as many factors as apply to your **pre-COVID-19** consultation approach.

- ☐ Pre-qualifying education / training
- ☐ Pre-qualifying clinical experience(s)
- ☐ Post-qualifying / postgraduate education (e.g., PG Certificate or Diploma, Masters)
- ☐ Post-qualifying training (e.g., CPD seminars, short courses and/or workshops)
- ☐ Post-qualifying clinical experience(s)
- ☐ Clinical guidelines
- ☐ Professional registrations / memberships
- ☐ Professional Indemnity insurance policies
- ☐ Workplace Code of Conduct
- ☐ Current research and/or Evidence-Based Practice (EBP)
- ☐ Mentorship and/or clinical supervision
- ☐ Other

a. If you selected Other, please specify:

*22.***Please elaborate** on your personal interaction style or consultation approach if you have additional comments. *Optional*

*23.*On a scale ranging from **1 (not at all)** to **5 (very large extent)**, please indicate to what **extent the COVID-19 pandemic has impacted your consultation approach** for patients with chronic or persistent LBP. *Required*

Please select at least 1 answer.
You may select up to 3 answer(s) (e.g., '4 - Large extent' and 'Currently unable to practice').

	**1 – Not at all**	**2 – Small extent**	**3 – Moderate extent**	**4 – Large extent**	**5 – Very large extent**	**Unsure**	**Currently unable to practice**	**Have not treated patients with chronic LBP**
Impact of COVID-19 on your consultation approach	☐	☐	☐	☐	☐	☐	☐	☐

a. **Please elaborate** on your response if you have additional comments.

Research Findings

If you are interested in receiving a **summary of the findings**, please include your **email address** in the textbox below:

*24.*Please **DO NOT** provide your **NHS** email address: *Optional*
Please enter a valid email address.

- Your email address will be securely stored on this password protected online survey platform (**hosted by Jisc**: https://www.onlinesurveys.ac.uk/).
- Jisc acts as the Data Processor, and is both GDPR compliant and ISO 27001 certified.
- Once the summary of findings are sent out, your email address will be confidentially destroyed.
- Only Bronwyn Sherriff (primary researcher) has direct access to the raw (non-anonymised) survey responses collected via Jisc.
- Any personal or identifying information will be removed, prior to such data being exported from the Jisc platform or being shared.

Please click the 'Finish' button to submit your responses, otherwise, they will not be saved.

Page 10: Thank you

- **Thank you** for choosing to take part in this Delphi-study.
- I am truly **grateful for your time** and invaluable **insights**.

Please do not hesitate to contact me if you have any queries or would like to be kept updated on the findings of this study.

Ms Bronwyn Sherriff: bsherriff@bournemouth.ac.uk
Alternatively, you are welcome to contact one of my PhD supervisors instead:

- Prof. Carol Clark: cclark@bournemouth.ac.uk
- Prof. David Newell: dnewell@aecc.ac.uk
- Dr Clare Killingback: c.killingback@hull.ac.uk
